# The genomics of ecological vicariance in threespine stickleback fish

**DOI:** 10.1038/ncomms9767

**Published:** 2015-11-10

**Authors:** Marius Roesti, Benjamin Kueng, Dario Moser, Daniel Berner

**Affiliations:** 1 Zoological Institute, University of Basel, Vesalgasse 1, Basel 4051, Switzerland

## Abstract

Populations occurring in similar habitats and displaying similar phenotypes are increasingly used to explore parallel evolution at the molecular level. This generally ignores the possibility that parallel evolution can be mimicked by the fragmentation of an ancestral population followed by genetic exchange with ecologically different populations. Here we demonstrate such an ecological vicariance scenario in multiple stream populations of threespine stickleback fish divergent from a single adjacent lake population. On the basis of demographic and population genomic analyses, we infer the initial spread of a stream-adapted ancestor followed by the emergence of a lake-adapted population, that selective sweeps have occurred mainly in the lake population, that adaptive lake–stream divergence is maintained in the face of gene flow from the lake into the streams, and that this divergence involves major inversion polymorphisms also important to marine-freshwater stickleback divergence. Overall, our study highlights the need for a robust understanding of the demographic and selective history in evolutionary investigations.

## Abstract (web summary)

Threespine stickleback fish are adapted to lake and stream habitats in Central Europe. Here, the authors show colonization of a lake basin by a stream-adapted ancestor, followed by the emergence of a lake-adapted population in the face of gene flow across lake–stream boundaries.

## Graphical abstract

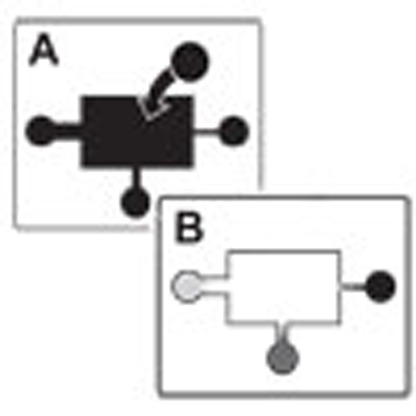

Parallel (or convergent^1^) phenotypic evolution—that is, the repeated independent emergence of a specific phenotype associated with a specific habitat, can provide important insights into the determinism of natural selection. The reason is that similar phenotypes are unlikely to evolve repeatedly in association with an environment by chance. An aspect of parallel evolution now made amenable to investigation through advances in molecular techniques is to what extent the repeated evolution of similar phenotypes involves the same genetic loci^1^,^2^,^3^. A common analytical framework adopted to address this question is to compare multiple population pairs, each believed to represent an independent replicate of adaptive population divergence between two ecologically different habitats. The evolutionary independence of these population pairs is generally established by demonstrating that the genetic relatedness between the populations within pairs, as inferred from markers little influenced by selection (for simplicity hereafter called ‘neutral markers'), exceeds that seen among the pairs. If so, the population pairs are assumed to represent replicates of independent ecological divergence and are screened for genomic loci exhibiting signatures of divergent selection between the habitats (for example, high divergence relative to some genome-wide baseline). Finally, the resulting lists of such loci are compared to draw conclusions about the extent of parallel evolution at the genomic level (for example, refs 4, 5, 6, 7, 8, 9; for closely related inferential approaches see refs 10, 11, 12).

A possibility rarely considered in such investigations is that the demographic and selective history of the study populations may complicate or preclude inferences about parallel evolution. Such a situation occurs when multiple patches of two ecologically different habitats are initially colonized by a single ancestor already adapted to one habitat type. Subsequently, local adaptation in the alternative habitat drives ecologically based reproductive isolation between the habitats, although some genetic exchange across habitat boundaries will continue in the absence of absolute geographic barriers. The outcome of such ‘ecological vicariance'^13^ with genetic exchange will mimic parallel evolution^14^. The reason is that gene flow between ecologically different populations in contact will cause genetic differentiation at neutral markers to be lower within than among population pairs—the pattern also expected under parallel divergence. Moreover, under both scenarios, loci under divergent selection will be relatively protected from exchange between the populations in contact and can therefore maintain stronger differentiation between the habitats than neutral loci^12^,^15^,^16^,^17^. In situations involving ecological vicariance with gene flow, comparing multiple population pairs can permit the reliable identification of selected loci and thus confirm divergent selection, but inference about the genetic basis of independent parallel evolution will be inappropriate because divergence did not occur repeatedly.

Distinguishing parallel divergence from ecological vicariance scenarios is thus crucial when attempting to explore how deterministically selection acts at the genomic level during evolution. While this distinction is not possible based on phylogenetic relationships at neutral markers^18^,^19^, it can be achieved by combining thorough analyses of molecular signatures around the loci under divergent selection with robust reconstructions of the populations' demographic history^14^,^20^. We here present such an investigation based on populations of threespine stickleback fish (*Gasterosteus aculeatus*) adapted to lake and stream habitats within the Lake Constance basin in Central Europe.

This stickleback system comprises a large and genetically well-mixed population residing in Lake Constance—with 571 km^2^ the third largest lake in Central Europe—and multiple adjoining stream-resident populations inhabiting the lake's tributaries^21^,^22^,^23^. The lake and stream habitats are ecologically different, as mirrored by the lifestyles of the stickleback populations: lake fish forage pelagically (that is, in the open water) on zooplankton, whereas the stream populations feed on benthic (substrate-dwelling) macroinvertebrates. This different resource use is paralleled by divergence in foraging morphology and life history^21^,^23^,^24^. Lake and stream populations in the Lake Constance basin also differ predictably in their extent of lateral plating^21^,^23^. Just like marine stickleback^25^, pelagic Lake Constance fish exhibit a series of bony plates covering their entire flank, providing protection from vertebrate predators in the open water^26^. By contrast, multiple stream populations show a reduction in the extent of lateral plating, the phenotype predominant in freshwater stickleback on a global scale.

Although the Lake Constance stickleback system has certainly formed postglacially (that is, within the last 12,000 years^27^), its origin is not resolved. One view is that a human introduction during the nineteenth century initially led to the establishment of a large lake population, and that subsequently multiple stream populations diverged independently from the lake population^21^,^23^. This scenario thus implies parallel divergence. An alternative is a more ancient natural colonization of the Lake Constance region by an already stream-adapted ancestral population from the Danube drainage^23^ (now draining into the Black Sea, hence disconnected from the Lake Constance basin), providing the potential for an ecological vicariance scenario.

The first goal of our study is to combine multiple lines of molecular evidence, based on dense genome-wide single-nucleotide polymorphisms (SNPs) obtained through restriction site-associated (RAD) sequencing^28^, to resolve the demographic and selective history of lake–stream divergence in the Lake Constance stickleback system. We demonstrate that adaptive divergence has occurred in the face of gene flow in an unexpected historical context, pointing to limitations in the standard interpretation of repeated phenotypic evolution. Based on these insights, we then dissect the molecular consequences of divergent selection in target regions, including the prime locus underlying divergence in lateral plating, and finally examine the role of chromosomal inversions in adaptive divergence.

## Results and Discussion

### Demography and population genomic analyses

**Figure 1:**
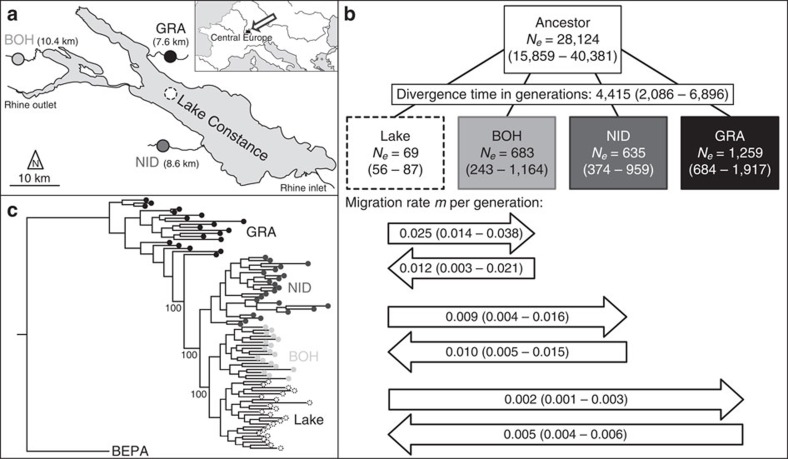
Geographic context, demography and phylogeny of the study populations. (**a**) Location of the study populations from the Lake Constance basin, including the panmictic lake population and the three tributary stream populations (BOH, NID and GRA; the same colour coding and line types identifying the populations are used throughout the paper). Numbers in parentheses indicate the water distance between each stream site and the lake. (**b**) Estimated age of the split of the study populations from their common ancestor (divergence time), effective population sizes (numbers within boxes), and bi-directional migration rates between the lake and each stream population (numbers in horizontal arrows, representing the long-term proportion of immigration into the target population from the source population per generation forward in time). The values are based on an estimated SNP mutation rate of 6.8 × 10^−8^. Numbers in parentheses are 95% bootstrap confidence intervals. (**c**) Phylogenetic relationship among the study populations visualized by a maximum likelihood tree rooted using a North American stickleback (BEPA, Bear Paw Lake, Alaska) as outgroup. Bootstrap support in per cent is given for the key nodes.

Our investigation focuses on four stickleback populations, including the panmictic (Supplementary Fig. 1) Lake Constance population (hereafter simply ‘lake') and three stream populations residing in tributaries (referred to as Bohlingen (BOH), Nideraach (NID) and Grasbeuren (GRA); see also refs 21, 23) (Fig. 1a), each represented by 22–25 individuals. To reconstruct the demographic history of these populations, we parameterized a divergence with gene flow model by using coalescent simulations based on the populations' joint allele frequency spectra^29^ derived from 14.8 million nucleotide positions on 166,711 RAD loci across the 460-Mb stickleback genome. This analysis indicated that the study populations—exhibiting relatively small estimated effective population sizes (extremely small in the lake, largest in GRA)—split from an at least 20 times larger ancestral population a few thousand generations (and years, since the typical life span of stickleback in this system is 1–2 years^23^,^24^) ago (Fig. 1b). Qualitatively similar estimates were obtained with an alternative model including only two stream populations (Supplementary Fig. 2). Also, long-term rates of lake–stream gene flow differed approximately tenfold, being highest between the lake and the BOH population, and lowest between the lake and the GRA population.

Next, we compared population-specific allele frequency spectra and found that across almost all minor allele frequency (MAF) classes, the lake exhibited the lowest and GRA the highest number of polymorphisms, with BOH and NID being intermediate (Supplementary Fig. 3). The lake also displayed the highest proportion of monomorphic SNPs, and the lowest proportion of tri-allelic SNPs (Supplementary Table 1). These findings clearly demonstrate that genetic diversity is lowest in the lake and increases from BOH to NID to GRA. Moreover, because the divergence among our study populations is recent (Fig. 1b, Supplementary Fig. 2) and the sharing of polymorphisms is extensive (Supplementary Table 1), most of the genetic variation in the present populations must have been standing in their common ancestor.

Calculating genome-wide baseline differentiation (that is, median *F*_ST_) for each of the three lake–stream pairings revealed an increase in population differentiation from 0.005 in the lake–BOH comparison to 0.013 and 0.061 in the lake–NID and lake–GRA comparisons, whereas no stream–stream population comparison yielded baseline *F*_ST_ higher than 0.056 (BOH–GRA; NID–GRA: 0.047; BOH–NID: 0.012). In a rooted phylogeny, the lake population emerged as a distal branch nested within the more basal stream fish (Fig. 1c, Supplementary Fig. 4). An unrooted phylogeny further confirmed the close relatedness of the lake and BOH populations and the lower genetic diversity in the lake than in the streams (Supplementary Fig. 5).

**Figure 2:**
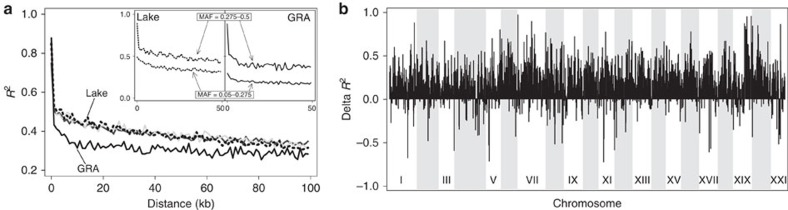
Linkage disequilibrium across the stickleback genome. (**a**) Magnitude of LD (squared correlation of allele frequencies) between SNP pairs in relation to their distance on a chromosome, shown for the lake (dotted black line), BOH (solid light grey line), NID (solid dark grey line) and GRA (solid black line) population. The main panel uses a minimal MAF threshold of 0.05. The insert panels display LD separately for low-MAF (0.05–0.275) and high-MAF (0.275–0.5) SNPs in the lake and GRA population. (**b**) Difference in LD between the lake and GRA population along the genome. The data points represent the average LD in the lake minus the average LD in GRA across non-overlapping 200-kb chromosome windows, yielding a measure called Delta *R*^2^.

Finally, we quantified linkage disequilibrium (LD) between all pairwise combinations of SNPs within all chromosomes in each population and found that strong allelic associations between SNPs occurred only over a scale of 1 kb or less; beyond this distance, LD was much weaker (Fig. 2a). The peak in LD at the smallest physical scale was driven by those SNPs exhibiting a high MAF; low-MAF SNPs exhibited more homogeneous and generally weaker LD at all distances (Fig. 2a, insert). Another striking result was that the extent of LD across the genome was substantially greater in the lake population (and the two stream populations little divergent from the lake, that is, BOH and NID) than in GRA. A similar result was obtained by exploring average LD among marker pairs within non-overlapping chromosome windows: across most of the genome, LD was much stronger in the lake than in GRA (Fig. 2b), a result insensitive to the MAF threshold (Supplementary Fig. 6). Finally, the similarity in the local magnitude of linkage across the genome between the lake and each stream population, expressed as the correlation of LD between the chromosome windows, declined from the lake–BOH (*r*=0.17) to the lake–NID (*r*=0.15) and the lake–GRA pairing (*r*=0.12) (all *P*<0.001).

In combination, the above analyses resolve the demographic and selective history of stickleback in the Lake Constance basin. First, the demography is inconsistent with the view that the populations originate from a recent introduction of (presumably few) founder individuals, and instead supports an earlier postglacial and extensive natural colonization, presumably via the Danube drainage^23^. Second, the demographic estimates of effective population size and all metrics of genetic variation make clear that the stream populations—and not the lake—represent the main reservoirs of genetic variation. This result is unexpected because Lake Constance is very large, and even conservative estimates of the present census size of its stickleback population range in the millions (personal communications from fishermen and fisheries authorities), which is certainly much greater than the size of any single stream population. (The streams investigated here are small, with an approximate average depth and width of 0.5 and 4 metres) Third, we observe the strongest genome-wide differentiation (*F*_ST_) between a stream and the adjoining lake population, and not in any of the comparisons between the stream populations separated by dozens of kilometres of lake habitat. Fourth, the lake population proves to be phylogenetically derived from stream fish. All these observations can be brought in line by the biogeographically plausible perspective that the Lake Constance basin was initially colonized by ancestral stream-adapted stickleback. This colonization gave rise to multiple stream-resident populations isolated from each other by the adjoining, ecologically different lake habitat—that is, an ecological vicariance scenario. Subsequently, the lake fish started to adapt to their novel habitat and thereby experienced strong genome-wide selection. This selection should not only have reduced genetic variation in the lake relative to the streams, but also have driven relatively elevated LD within the lake, predictions clearly borne out by our analyses.

**Figure 3:**
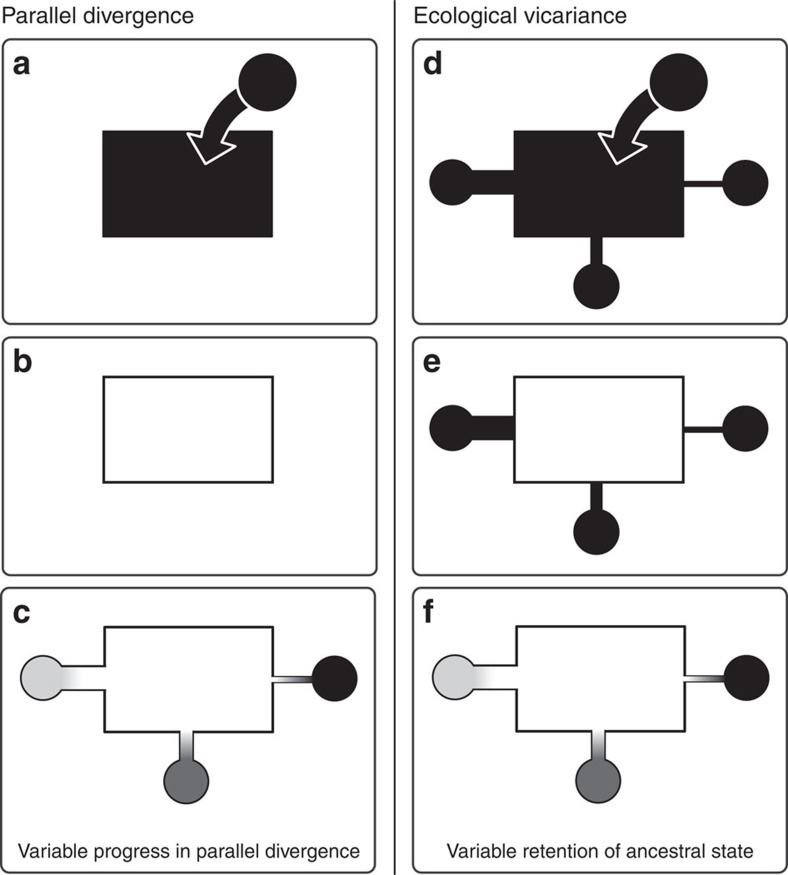
Alternative demographic scenarios explaining repeated population divergence. The alternatives are exemplified by multiple stream populations divergent from the adjacent lake population in the Lake Constance basin. In the ‘parallel divergence' scenario (panels **a**–**c**), a (stream-adapted) ancestor enters the lake (**a**) and becomes locally adapted (**b**). Subsequently, multiple stream populations derive independently via parallel evolution from the lake population (**c**), the latter thus representing their most recent common ancestor. The magnitude of lake–stream divergence in (**c**) (visualized as different grey shades) is determined by a combination of the time since colonization of each stream, the strength of local selection within each stream, and the extent of homogenizing gene flow from the lake into each stream. In this scenario, genetic variation available to local adaptation in the streams has been filtered during the adaptation of the lake population. Predictions here include greater genetic diversity in the lake than the stream populations, that *F*_ST_ is highest in stream–stream as opposed to lake–stream comparisons (due to founder events and relatively strong drift in these small populations), and that LD is highest in the streams (due to selective sweeps during adaptive divergence from the lake). In contrast, the ‘ecological vicariance' scenario (panels **d**–**f**) involves the colonization of the entire study region by an already stream-adapted ancestor (**d**), followed by local adaptation in the lake (**e**). The magnitude of lake–stream divergence is then primarily determined by the extent to which the stream populations can maintain their genetic integrity in the face of gene flow from the large lake population (**f**). Predictions here include greater genetic diversity in the streams than in the lake, highest *F*_ST_ in lake–stream as opposed to stream–stream comparisons, and strongest LD in the lake due to extensive selection. All these latter predictions are confirmed by our analyses.

A key implication of this ecological vicariance scenario (visualized in Fig. 3d–f) is that the stream populations cannot be considered independent products of parallel divergence from an ancestral lake population. The stream fish are closer to the ancestral state while the lake population is the most derived. (Note that the phylogeny in Fig. 1c also rules out the possibility that the lake population results from a secondary colonization; in this case, the lake fish would branch basally from the stream populations.) Variation in the magnitude of genetic and phenotypic lake–stream divergence thus reflects different levels of homogenizing gene flow (that is, introgressive hybridization) from the large lake to the stream populations rather than variable progress in repeated parallel divergence (Fig. 3a–c). Supporting this view, typical lake phenotypes can sometimes be found at our BOH stream sample site during the breeding season (personal communication from fishermen). This highlights the potential for extensive genetic exchange in the one lake–stream pairing also exhibiting the highest migration rate estimates and the lowest genetic differentiation.

The strong genome-wide footprint of selection in the lake population, observed as relatively reduced genetic diversity and elevated LD, also raises an important methodological caveat. Marker-based approaches to demographic inference generally assume that allele frequencies reflect selectively neutral processes^29^,^30^,^31^. In our study, the reduction of genetic variation by widespread selection in the lake clearly dissociates marker-based estimates of effective population size from biologically plausible census population sizes; the lake population, and to a lesser extent also the two stream populations strongly influenced by gene flow from the lake (BOH and NID), certainly have their estimated effective population sizes biased downward relative to the GRA stream population. This highlights the benefit of backing up genetic inferences of demography with analyses of the selective history and with qualitative information from the field.

### Genomically localized characterization of selection

**Figure 4:**
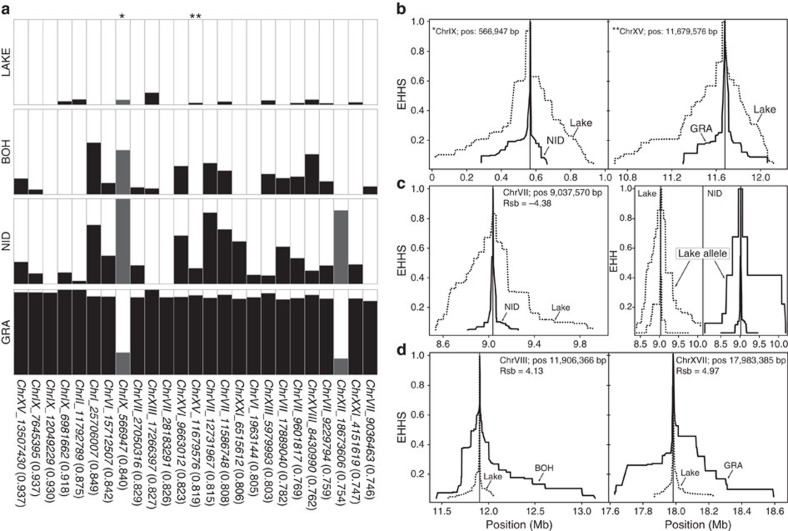
Localized signatures of selection. (**a**) Allele frequencies within each population at the 25 *F*_ST_ extremes (columns). The proportion of the SNP alleles predominant in the lake are shown in white, while the proportion of the alleles predominant in the streams are either black (when the extreme *F*_ST_ value emerged from the lake–GRA genome scan, *N*=23) or dark grey (extreme *F*_ST_ value observed in the lake–NID scan, *N*=2). On the bottom, the genomic position and the highest *F*_ST_ value observed across all lake–stream comparisons are given for each *F*_ST_ extreme. (**b**) Lake and stream haplotype decay (EHHS) around representative *F*_ST_ extremes (flagged by asterisks in **a**) identified in the lake–NID *F*_ST_ scan (left panel) and in the lake–GRA scan (right panel). (**c**) Haplotype decay in the lake and the NID stream population around a representative negative Rsb extreme identified in the lake–NID Rsb scan (left panel). For the same Rsb extreme, the right panel displays allele-specific haplotype decay (EHH) around each allele within each population separately (both alleles occur in both populations; the allele predominant in the lake is labelled ‘Lake allele'). (**d**) Lake and stream haplotype decay around two representative positive Rsb extremes identified in the lake–BOH Rsb scan (left panel) and in the lake–GRA scan (right panel). Note that the scale of the *x* axis varies in **b**–**d**.

The above genome-wide analyses indicated that the lake population has been particularly strongly influenced by widespread selective sweeps. To confirm this asymmetry in selection at a finer scale, we inspected localized signatures of selection at two classes of loci within the genome. The first, called *F*_ST_ extremes, included the 25 independent SNPs displaying the strongest lake–stream differentiation across all three lake–stream *F*_ST_ scans combined (79,770 total SNPs). None of these extreme SNPs showed fixed allelic differences between the habitats, but nearly so: *F*_ST_ ranged from 0.94 to 0.75—remarkably high values given the low baseline differentiation (Fig. 4a; genome-wide *F*_ST_ profiles visualizing the strikingly heterogeneous genomic divergence in all three lake–stream comparisons are provided in Supplementary Fig. 7). The *F*_ST_ extremes were found on 11 different chromosomes and derived mostly from the lake–GRA comparison that also produced the greatest baseline differentiation. Inspecting allele frequencies at the *F*_ST_ extremes showed that the MAF was generally lower in the lake population (14 out of the 25 SNPs were monomorphic) than in the corresponding stream population (with only four monomorphic SNPs; binomial test for similar occurrence of monomorphic SNPs: *P*=0.007; Fig. 4a), suggesting that selection has mainly occurred, or has been more effective, in the lake. At the *F*_ST_ extremes, those alleles near fixation in one of the stream populations were generally also present in the other stream populations, with the frequency of these stream alleles increasing from BOH to NID to GRA (Fig. 4a). Finally, we found that haplotype decay around the *F*_ST_ extremes was slower in the lake than in the focal stream population (binomial *P*=0.004; Fig. 4b).

The *F*_ST_ extremes represented genomic regions with nearly complete lake–stream allele frequency divergence, hence reflecting strong selection. To search for weaker or ongoing selective sweeps, we delimited a second class of loci based on haplotype structure^32^,^33^. Specifically, we used Rsb^34^ to compare the rate of haplotype decay between the lake and the streams at 87,738 SNPs for each lake–stream comparison. Following the convention that positions with an absolute Rsb value >4 provide compelling evidence of selection (for example, ref. 35), we identified a total of 22 such ‘Rsb extremes' on 11 chromosomes across all three lake–stream comparisons (lake–stream Rsb profiles are presented as Supplementary Fig. 8; in contrast to the *F*_ST_ extremes, Rsb extremes emerged from all lake–stream contrasts, Supplementary Fig. 9). Interestingly, examining allele-specific haplotype structure revealed that within both habitats, the lake alleles were surrounded by relatively longer haplotype tracts than the alternative stream alleles (Fig. 4c). This indicates that alleles selected positively in the lake, but presumably negatively in the streams, are maintained at substantial frequency in the streams by gene flow from the lake population. Finally, our haplotype-based analysis also revealed signatures of selective sweeps that have occurred in the stream habitat (Fig. 4d, Supplementary Fig. 9).

Overall, our analyses of localized signatures of selection provide strong support for the selective scenario indicated by the genome-wide signatures: selection is wide-spread across the genome and is asymmetric, with more extensive sweeps having occurred in the lake than in the stream populations. Moreover, lake–stream divergence in the Lake Constance basin has clearly occurred in the face of gene flow. Consistent with the census size (but not the estimated effective population size) of the Lake Constance population being orders of magnitude larger than the stream populations, introgression occurs primarily from the lake into the streams. Nevertheless, many loci resist gene flow and maintain substantial differentiation from the lake^12^,^15^,^16^,^17^, thereby generating heterogeneous genomic divergence between the lake and the stream populations^36^.

### Signatures of selection around a known adaptation locus

**Figure 5:**
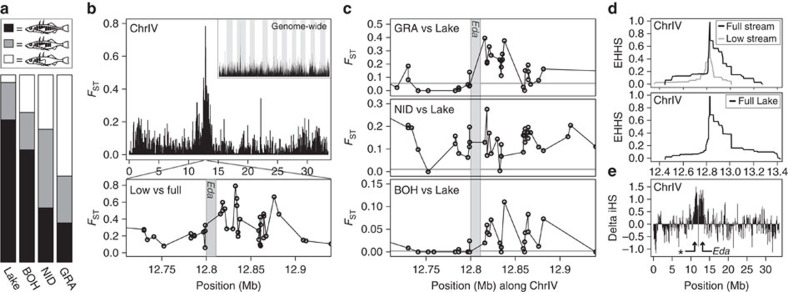
Lake–stream divergence in lateral plating and the associated molecular signatures. (**a**) Frequency of the three plate phenotypes (completely, partially and low-plated) in the four study populations. (**b**) Genetic differentiation (*F*_ST_) between completely and low-plated stream stickleback reveals a peak on chromosome IV, with the highest *F*_ST_ value immediately downstream of the *Eda* coding region (grey vertical bar). (**c**) Divergence (*F*_ST_) profiles around the plate locus for all lake–stream comparisons, with the horizontal grey lines indicating baseline differentiation (note that the scale of the *y* axes varies). (**d**) Haplotype decay (EHHS) in completely and low-plated stream stickleback around the *Eda*-associated SNP exhibiting the strongest phenotype–genotype association in the bulk segregant analysis (see **b**, bottom) (upper panel). The lower panel shows haplotype decay around the completely plated allele at the same SNP in the lake population. (**e**) Profile of the difference in the rate of haplotype decay between completely and low-plated stream stickleback along chromosome IV. High positive values of this ‘Delta iHS' metric, indicating more extensive haplotype tracts in completely plated individuals, occur across a broad region centred at the *Eda* locus, and in a second genomic region nearby (indicated by an asterisk; note the corresponding *F*_ST_ peak in the upper panel of **b**).

Our analyses of localized signatures of selection within the genome focused on regions likely important to adaptation to the lake and stream habitats, yet it is unknown what phenotypes the polymorphisms in these regions influence. For the extent of lateral plating, however, it was possible to take an alternative route and to investigate the molecular signatures produced by selection on a trait known *a priori* to be important to lake–stream divergence. We started at the phenotypic level by establishing that lake individuals were mostly completely plated, whereas plating was relatively reduced in all stream populations, most clearly so in NID and GRA (lake–BOH permutation test for similar plating: *P*=0.420; lake–NID: *P*=0.002; lake–GRA: *P*<0.001) (Fig. 5a). This agrees with earlier work using different populations and/or samples from the same basin^21^,^23^. Next, we performed a bulk segregant analysis (BSA) by pooling all completely and all low-plated stream fish into two separate groups. Genetic differentiation between these groups across genome-wide SNPs revealed a region on chromosome four (ChrIV) harbouring markers with a very strong association between allelic state and phenotype (Fig. 5b). The peak association (*F*_ST_=0.78) occurred immediately downstream of the Ectodysplasin (*Eda*) gene. This locus is known as major determinant of lateral plating^37^,^38^, with a causative *cis*-regulatory polymorphism having been identified 1 kb downstream of the coding region^39^. No SNP outside this region on ChrIV displayed *F*_ST_>0.38.

Combined, the phenotypic data and BSA indicate that differentiation in plating among our study populations involved adaptive lake–stream divergence at the *Eda* locus. We thus predicted molecular footprints of selection at this locus. To evaluate this prediction, we inspected all three lake–stream *F*_ST_ scans for the magnitude of differentiation around *Eda* (Fig. 5c). As expected from the plate morph distribution (Fig. 5a), the strongest differentiation occurred in the lake–GRA comparison (*F*_ST_=0.40), just 5.7 kb downstream of *Eda*. However, in this particular comparison, the most divergent SNP near *Eda* ranked only within the upper 3.5 percentile of the genome-wide *F*_ST_ distribution (lake–BOH and lake–NID comparisons: 8.8 and 2.3 percentile). Similarly, the highest absolute Rsb value around *Eda* (1.17) also emerged from the lake–GRA comparison but fell only within the upper 23 percentile of the genome-wide Rsb distribution. Hence, thousands of SNPs displayed a stronger deviation from selective neutrality than the *Eda* locus. Accordingly, subjecting the lake–GRA pairing to a standard selection outlier detection analysis (*BayeScan*^40^) failed to provide any evidence of selection at SNPs surrounding *Eda* (Supplementary Fig. 10).

More nuanced insights into the evolution of lateral plating were obtained by analysing haplotype structure around the *Eda* locus: in the streams, where both *Eda* alleles (still) occur at substantial frequencies, haplotype decay was slower around the allele associated with complete plating (Fig. 5d, top). Moreover, the haplotype structure around the completely plated allele in the streams matched the haplotype structure around this allele in the lake (where the low-plated allele was too rare to characterize LD) (Fig. 5d, bottom). Together, this indicates that selection for complete plating in the lake has been more effective than selection against plates in the streams, and again suggests the maintenance of an unfavourable variant—and the associated phenotype—in the streams by gene flow from the lake (see also Fig. 4c). To fully appreciate the extent of LD driven by selection on lateral plating, we again took a bulk segregant approach by treating all completely and low-plated stream fish as separate groups, and looked for distortions between these groups in the rate of haplotype decay along ChrIV. This confirmed that selection on the *Eda* variant driving complete plating has been much more intense than selection on the low-plated variant, and showed that the associated sweep has influenced haplotype structure at the scale of megabases (Fig. 5e). Unexpectedly, this scan also detected a second, similarly strong selective sweep in completely plated stickleback centred at 11.4 Mb. This latter region also exhibited a clear signature of divergence in the *F*_ST_-based BSA (Fig. 5b, top): the differentiation peak in this region (*F*_ST_=0.31) fell within the top 0.06 per cent of the genome-wide distribution.

Together, the investigations at the *Eda* locus highlight our limited ability to elucidate the genetic basis of adaptive population divergence based on genetic markers when selective sweeps are incomplete. Neither the magnitude of differentiation (*F*_ST_) nor haplotype structure (Rsb) among populations allowed the major plate locus to emerge as an obvious selection candidate—despite substantial evolution in the associated ecologically important phenotype, and despite an extensive selective sweep visible when comparing haplotype structure among individuals grouped by phenotype. Given that stronger signatures than those around *Eda* are numerous in our data sets, we conclude that hundreds of genomic regions must be involved in the adaptive divergence into lake and stream habitats. We further propose that lateral plate evolution in the Lake Constance basin is governed by at least one other locus besides *Eda*. Inspecting the newly detected region on ChrIV indeed produces a strong candidate gene, *Col23a1* (bp-position 11,443,468–11,468,190; this specific segment contained the highest-*F*_ST_ SNP observed across the new candidate region in the BSA). Like *Eda*, this gene encodes a transmembrane collagen involved in the development of the epidermis^41^. Since the new candidate region and *Eda* occur in close proximity (c. 1.4 Mb apart) in a low-recombination chromosome region^42^, it is tempting to speculate that the coupling of alleles in the two regions might facilitate divergence in plating relative to the situation where each locus segregates independently^12^,^43^.

### Detection and characterization of inversions

Our genetic data indicate that lake–stream divergence in the Lake Constance basin has occurred in the face of gene flow. Genetic polymorphisms predicted by theory to resist homogenizing gene flow and to diverge between populations particularly well are chromosomal inversions^43^,^44^,^45^. The reason is that different inversion types can physically couple alleles promoting adaptation to different habitats across multiple loci. The integrity of these allele clusters is easily maintained, because a single crossover within the inversion generally produces unbalanced meiotic products in inversion heterozygotes (that is, heterokaryotypes), thus effectively suppressing recombination^46^,^47^. Consequently, alternative inversion types can be considered single large-effect alleles.

**Figure 6:**
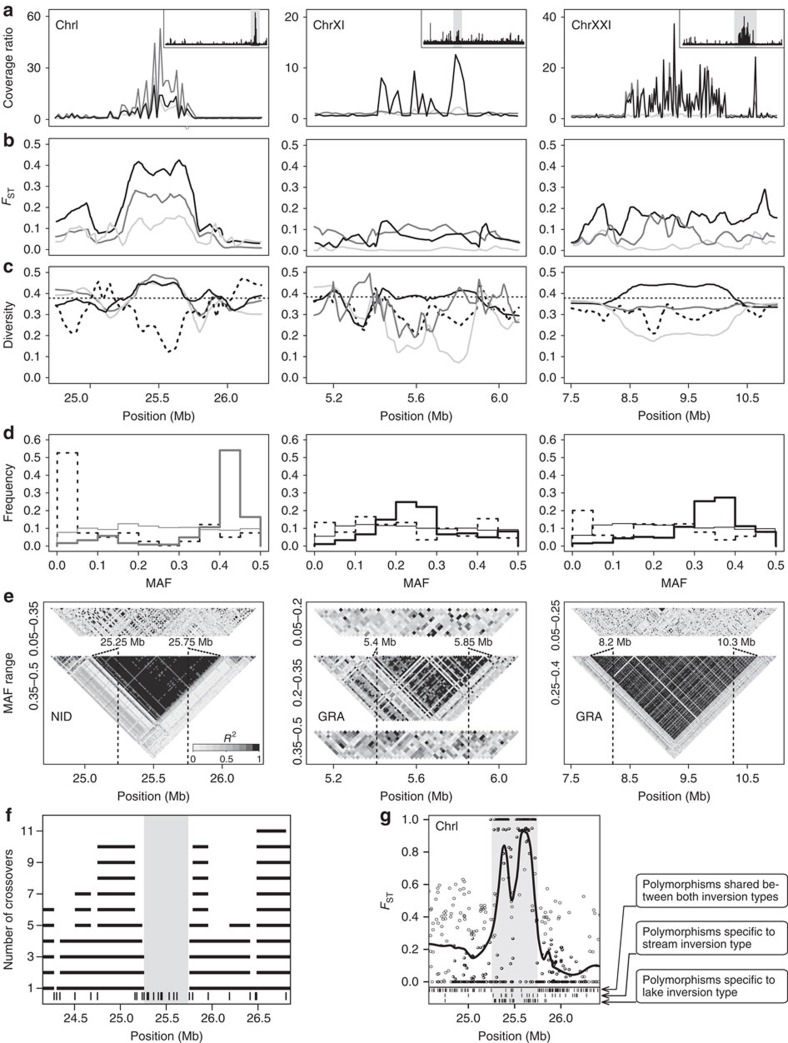
Detecting and characterizing large chromosomal inversions in lake and stream stickleback. (**a**) RAD sequence coverage of the stream populations relative to the lake population (coverage ratio), shown separately for each lake–stream population pair (lake–BOH light grey, lake–NID dark grey and lake–GRA black) along focal segments of the chromosomes I, XI and XXI harbouring an inversion. The inserts show the coverage ratio along the entire chromosomes (focal segments shaded grey), based on the coverage data pooled across the three stream populations. (**b**) Genetic differentiation between the lake and each stream population, and (**c**) allelic diversity at SNPs within each of the four populations, around the three inversions. (**d**) MAF distribution for SNPs located within each inversion, shown for the stream population exhibiting the strongest coverage ratio distortion relative to the lake (see **a**) (thick solid line). For comparison, the genome-wide MAF distribution for the same stream population (thin solid line), and the MAF distribution for all inversion SNPs in the lake population (dotted line) are also plotted. (**e**) Linkage disequilibrium heat maps based on SNPs from distinct MAF classes shown for the same stream populations as in **d**. (**f**) Recombination between the ChrI inversion types (the inverted segment is shaded grey) in a laboratory cross. Black horizontal bars indicate the number of crossovers observed between two neighbouring markers (vertical bars on the bottom), and the grey profile shows the corresponding recombination rate. (**g**) Genetic differentiation (raw values and smoothed profile) between individual pools of ChrI inversion homozygotes. The bottom part indicates the position of SNPs shared between the two inversion types, and of those unique to each.

To test this idea, we examined if lake–stream divergence in the Lake Constance basin was promoted by chromosomal inversions. For this, we scanned the genome for extended distortions in the relative RAD sequence coverage between the lake and each stream population (Supplementary Fig. 11). This produced three strong candidates, located on ChrI (approximate length: 500 kb), ChrXI (450 kb) and ChrXXI (2.1 Mb) (Fig. 6a)—all coinciding with inversions recently identified in a comparison of marine and freshwater stickleback^11^. For two of these candidate inversions (ChrI and ChrXI), we designed PCR primers across expected inversion breakpoints based on our RAD sequences, and the presence/absence of PCR products confirmed that these regions were inversions (Supplementary Fig. 12). We then performed several complementary analyses to characterize the three inversion polymorphisms in our populations. Inspecting inversion-specific allele frequencies revealed that the lake population was consistently fixed for one inversion type, whereas the stream populations were polymorphic at two (NID) or all three inversions (BOH and GRA). However, only at the ChrI inversion were lake–stream frequency shifts strong enough to drive clearly elevated *F*_ST_ relative to baseline differentiation (Fig. 6b). Consistent with only the stream populations being polymorphic for the inversions, the allelic diversity at polymorphic sites within the inversions tended to be elevated in the stream populations relative to the lake (Fig. 6c). However, the segregation of an inversion type at very low frequency within a population sometimes generated an excess of SNPs displaying reduced diversity relative to the genomic baseline within that population (BOH and NID at the ChrXI and ChrXXI inversions, Fig. 6c). The stream populations also exhibited a clear excess of SNPs falling into the specific MAF class mirroring the relative frequency of the minor inversion type (Fig. 6d). SNPs within this MAF class—but not those from other MAF classes—revealed extended blocks of nearly perfect LD caused by the inversion polymorphisms in the streams (Fig. 6e).

For the ChrI inversion, we experimentally confirmed suppressed recombination in inversion heterozygotes by inspecting crossover frequencies in an F2 intercross derived from two parental individuals homozygous for either inversion type^38^,^42^. Not a single crossover occurred within the inversion, but recombination immediately adjacent to the inversion was frequent (Fig. 6f; see Supplementary Fig. 13 for a negative control of this analysis). Nevertheless, for large inversions, theory predicts that occasional double crossovers should allow some genetic exchange between the inversion types, albeit not near the inversion breakpoints^47^,^48^. We examined this prediction for the ChrI inversion by comparing homozygotes for one inversion type to homozygotes for the other type, considering individuals from all populations. We found that while these two groups were fixed for different SNP alleles across most of the inversion, differentiation decayed in a narrow region in the centre of the inversion (Fig. 6g). This region was also relatively enriched for polymorphisms shared between the two inversion types, but contained relatively few SNPs unique to either of the two types (Fig. 6g, bottom).

**Figure 7:**
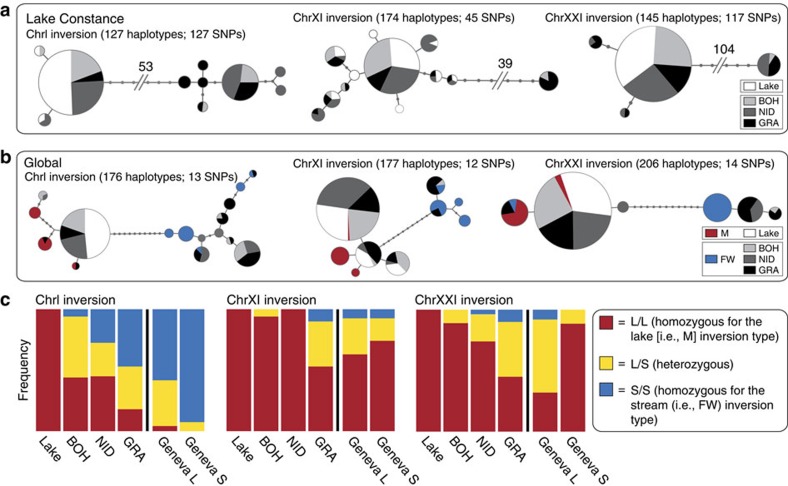
Phylogenies and habitat associations of the inversions. (**a**) Haplotype phylogenies restricted to SNPs located within the inversions, based on individuals from the Lake Constance basin. Pie sizes reflect the relative frequency of each haplotype, and internodes are mutational steps. Only haplotypes recovered more than twice are shown (total haplotype numbers are given above the networks). (**b**) Haplotype networks as in **a**, but additionally including individuals from marine and freshwater populations across the species' global range^11^. (**c**) Frequency of the three diploid genotype classes at each inversion in the four populations from the Lake Constance basin, and in the lake–stream pair from the Lake Geneva basin. The colour coding follows **b**.

To learn more about the history and ecology of the three inversion polymorphisms, we next established the phylogenetic relationship among our study individuals using haplotype information based on SNPs located within the inverted regions only. For each inversion, this revealed the presence of two haplotype clusters separated by a deep split (Fig. 7a). In line with our findings from the allele frequencies at putative loci under selection (Fig. 4a), Lake Constance fish consistently harboured haplotypes from one of these clusters only, whereas all stream populations contained haplotypes from both clusters. Repeating the phylogenetic analysis by including SNPs extracted from 21 previously sequenced marine and freshwater stickleback sampled across the species' global distribution^11^ produced a striking result: haplotypes representing the inversion type for which the Lake Constance population was fixed clustered consistently or were even identical with haplotypes recovered in marine stickleback (Fig. 7b). Conversely, haplotypes representing the inversion type found exclusively in the streams were closely related to, or identical with, haplotypes from global freshwater populations. To further explore how consistently these inversion polymorphisms are recruited for lake–stream divergence, we investigated SNP data for individuals sampled from Lake Geneva and from one of its tributary streams, waters documented to have been colonized by stickleback very recently (nineteenth century) and independently from the Lake Constance basin (see references in refs 21, 22; genome-wide divergence in this lake–stream pair is described in Supplementary Fig. 14). We here again recovered all three inversion polymorphisms (Fig. 7c, Supplementary Fig. 14). At the ChrI inversion, the direction of lake–stream divergence was congruent between the Lake Constance and Lake Geneva basins, whereas the ChrXI showed no divergence in the latter. Surprisingly, the direction of lake–stream divergence at the ChrXXI inversion was reversed between the two basins.

Overall, a first insight emerging from our analyses of inversions is that the relative frequencies of inversion types need to be taken into account when scanning population genomic data for the presence of such polymorphisms. Characteristic signatures like extended blocks of SNPs displaying exceptional levels of population differentiation or strong LD can become evident only when restricting SNPs to the appropriate MAF class. Second, our analysis of the ChrI inversion shows that genetic exchange between inversion types can occur despite effective overall recombination suppression, and that this exchange is biased towards the inversion centre. To our knowledge, this has previously been demonstrated only for much larger inversions in *Drosophila* and *Anopheles*^49^,^50^. Our data from the laboratory cross further suggest exceptionally high recombination rates in the collinear segments immediately flanking the inversion (Fig. 6f). This is unexpected—double crossover encompassing a single inversion breakpoint should produce unbalanced chromatids, hence one would predict relatively reduced recombination in these regions^47^.

Finally, the distribution of inversion haplotypes in the Lake Constance basin suggests divergent lake–stream selection on these chromosomal rearrangements. Specifically, the occurrence of shared haplotypes at both inversion types within multiple, presently unconnected stream populations, and the consistent presence of only a single inversion type in the lake, indicate particularly effective sorting of ancestral standing variation in the lake population. This reinforces our conclusion of asymmetric selection based on the genome-wide analyses and the inspection of *F*_ST_ and Rsb extremes, and supports the view that inversion polymorphisms are ecologically relevant^44^. (We note that we could not find any indication of intrinsic incompatibility or transmission disequilibrium between the inversion types, as their frequencies did not deviate from Hardy–Weinberg expectation in any inversion-population combination. Details not presented, but see Fig. 7c.)

All inversion haplotypes occurring within Lake Constance further coincide with haplotypes predominant in marine stickleback. This suggests the presence of shared selective features between the ocean and large lakes—possibly mediated by a pelagic lifestyle in both habitats (see ref. 51 for similar evidence from trout)—driving genuine parallel evolution at a much larger geographic scale than our focal lake–stream system. In any case, these inversions are not (only) relevant to saltwater–freshwater adaptation^11^. To further complicate functional conclusions, the ChrXXI inversion has diverged in opposed directions between lake and stream stickleback in the Lake Constance and the Lake Geneva systems. This unexpected trend is unlikely to arise from drift in the young Geneva system: among the 50 most extreme genome-wide *F*_ST_ values in this exceptionally weakly divergent lake–stream pair (genome-wide median *F*_ST_=0), 22 (44%) map to the ChrXXI inversion, including the top value observed overall (*F*_ST_=0.338) (Supplementary Fig. 14). This suggests intense selection on this inversion polymorphism in the Geneva system. However, given the great number of genes coupled by each inversion (∼24, 25 and 109 genes for the ChrI, ChrXI and ChrXXI inversions), dissecting the precise target(s) of selection in different ecological contexts will remain a serious challenge. Finally, the detected sharing of haplotypes between our study populations (derived from Atlantic ancestors) and worldwide stickleback populations (including Pacific-derived fish), along with the vast mutational differentiation observed between the inversion types (Fig. 6g and Fig. 7b), indicates that all three inversion polymorphisms must be ancient.

To summarize, a main goal of our study was to dissect the demographic and selective history of adaptive diversification in lake and stream stickleback populations within a single lake basin. Combining demographic inference with broad scale and localized analyses of genetic differentiation and diversity, linkage disequilibrium and haplotype structure within the genome allows us to reject a standard scenario of parallel divergence of multiple stream populations from a shared ancestral lake population (Fig. 3a–c). Instead, our results support a history of ecological vicariance with gene flow. This latter scenario involves the widespread colonization of the Lake Constance basin by a stream-adapted ancestor, the subsequent emergence of a derived lake–adapted population through intense selection of standing variation and sustained gene flow across the lake–stream boundaries (Fig. 3d–f). Consequently, different magnitudes of overall divergence among the lake–stream pairings, and heterogeneous lake–stream divergence across the genome, do not mirror how strongly gene flow from the lake has constrained the emergence of adaptation in the streams, but how effectively introgression from the lake has eroded initial stream adaptation. Our work thus underscores that investigations of patterns of divergence consistent with parallel evolution should consider an alternative—that is, the repeated retention of shared ancestral variation, and should be rooted in detailed knowledge about the demographic and selective history of populations^14^. Nevertheless, nested within a vicariance background, our investigation of inversion polymorphisms indicates the recycling of the same genetic variants for adaptive divergence in seemingly different ecological contexts, and hence real parallel evolution on a large geographic scale.

Furthermore, our finding of highly heterogeneous genomic divergence conflicts with the recent theoretical prediction that adaptive divergence in the face of gene flow involving selection on extensive standing variation should produce genome-wide reproductive isolation and therefore limit heterogeneity in genome divergence^52^. Given the numerous factors influencing adaptive divergence in natural populations, we believe that it will remain very difficult to predict how fast and to what extent heterogeneous genomic divergence should build up. However, our study clearly supports the notion that heterogeneity in genome divergence is promoted by sustained gene flow between young populations adapting to ecologically different environments (for example, ref. 53). We challenge the claim that such heterogeneity represents the divergence of populations after reproductive isolation has become complete^54^.

Finally, our study adds molecular evidence to the idea that chromosomal inversions promote adaptive divergence by acting as loci of large effect^44^. However, lake–stream stickleback divergence certainly also involves numerous loci not located within chromosomal rearrangements, and selection on some of these loci appears at least as strong as selection on the inversions. Determining the importance of inversions relative to other adaptive polymorphisms in evolutionary diversification remains an important empirical issue.

## Methods

### Stickleback samples and marker generation

Specimens from the Lake Constance population were sampled at two localities (Romanshorn, Switzerland, *N*=12, and Unteruhldingen, Germany, *N*=13; for geographic details see ref. 23). Genetic structure is absent at any scale within this large lake (Supplementary Fig. 1 and refs 21, 23), so the two lake samples were combined to a single ‘lake' pool for all analyses. Stream stickleback were sampled from three geographically well-separated tributaries connected through the lake only (Fig. 1a). The stream sites correspond to the Bohlingen (BOH, *N*=22), Nideraach (NID, *N*=24) and Grasbeuren (GRA, *N*=24) localities in ref. 23 (for details on all specimens see Supplementary Table 2). Natural dispersal barriers are absent in all streams, but low man-made dams have likely restricted gene flow from the lake to the NID and GRA sites over the last decades. All work in this study was approved by the Veterinary Office of the Canton of Basel-Stadt (permit number: 2383).

DNA was extracted from stickleback fin and muscle tissue using either a MagNA Pure LC278 extraction robot (Roche, Basel, Switzerland) with the tissue Isolation Kit II, or the DNeasy Blood & Tissue Kit (Qiagen, Valencia, USA). After an RNase treatment, the extracts were standardized to 18 ng μl^−1^ based on multiple NanoDrop photospectrometer readings (Thermo Scientific, Wilmington, USA), and used to generate RAD DNA libraries essentially following the protocol described in ref. 5. The main modification was that we used the Nsi1 enzyme for DNA restriction, exhibiting a 7.5 times higher recognition site density (that is, c. 164,000 sites across the 460-Mb stickleback genome) compared with the commonly used Sbf1 restriction enzyme. We prepared 12 total RAD libraries, each combining individually 5mer-barcoded DNA from seven or eight of the 95 total individuals. For final enrichment, we pooled six replicate PCRs per library to reduce amplification bias.

Each library was single-end sequenced with 100 cycles on a separate Illumina HighSeq2000 lane. Raw sequence reads were parsed by individual barcodes and aligned to the improved assembly^42^ of the threespine stickleback reference genome^11^ by using Novoalign v2.07.06 (http://www.novocraft.com/products/novoalign; sequencing and alignment statistics are provided in Supplementary Table 2). We enforced unique alignment, tolerating an equivalent of ∼8 high-quality mismatches or gaps (flags: -t236, -g40, -x15). Alignments were BAM-converted in Samtools v0.1.11 (ref. 55). For individual consensus genotyping, we first applied two effective filters to further exclude RAD loci located on repeated elements. First, loci were excluded if they displayed a read coverage exceeding three times the mean coverage across all loci within an individual. Second, if a RAD locus was polymorphic, it was excluded if the two dominant haplotypes failed to account for >70% of all reads.

Loci passing the above filters were subjected to consensus genotyping using a refinement of our earlier haplotype-based algorithm^5^, which has been demonstrated to perform highly accurately^56^. The main novelty was that instead of building genotypes quality-aware base-by-base, we discarded sequence quality and treated the entire read as the genotyping unit. A diploid genotype was called if the read coverage contributed by the two dominant haplotypes, or the total coverage for monomorphic loci (‘effective coverage'), was 15 or greater (median total coverage across all RAD loci and individuals was 38.5 × ). Because we observed in our previous work that the distribution of the two haplotypes for heterozygous loci was over-dispersed relative to the binomial expectation, we avoided distinguishing homozygote from heterozygote genotypes based on a theoretical distribution. Instead, a locus was considered heterozygous if the ratio of the second most frequent haplotype to the sum of the first and second was >0.25. Otherwise, a locus was considered homozygous. If the effective coverage was below 15 but at least two, we called a haploid genotype only, based on the dominant haplotype. Loci with single-read coverage were discarded. Inspection of the haplotype distribution at RAD loci showed that with our sequence data, this defensive algorithm maximized both the detection of truly heterozygote loci and the exclusion of polymorphisms reflecting technical artifacts (Supplementary Fig. 15). To create the raw SNP matrix for downstream analyses, we pooled the consensus genotypes across all populations and extracted a maximum of six SNPs per RAD site, provided the haploid consensus genotype coverage across all individuals and populations was at least 80 × .

### Demography and phylogenetics

To explore the evolutionary history of our four study populations, we reconstructed their demography using the coalescent simulator *fastsimcoal2.1* (ref. 29). As input, we computed the observed joint site frequency spectrum (SFS) for each of the six pairwise population combinations. For this, we first sampled at random exactly 30 haploid consensus genotypes per RAD locus from each population. Loci with sparser coverage and those harbouring more than two polymorphisms with an identical frequency of the less common allele (that is, the ‘minor allele frequency' (MAF)) across the last 30 positions were ignored. The latter excluded uninformative sequential pseudo-SNPs from RAD loci harbouring a micro-indel polymorphism, and hence ensured that only true SNPs were considered. Next, we counted the occurrence of the minor allele at each of the 89 positions per RAD locus in each population to populate the SFS. This considered both monomorphic positions and bi-allelic SNPs. For the latter, the minor allele was defined based on the pool of all four populations. If the MAF of a SNP was exactly 0.5, both alleles were treated as minor and entered the SFS, but with a weight of 0.5 only (personal recommendation by L. Excoffier). The resulting joint SFS were based on 14.837 million base positions on 166,711 RAD loci. We additionally computed all population-specific SFS with the same resolution.

Using the observed joint SFS, we then performed simulations with *fastsimcoal2.1* to estimate the most likely parameter values for an evolutionary scenario in which the four focal populations split under gene flow from an ancestral population colonizing the Lake Constance basin. We here assumed that the populations in the different habitats established rapidly, justifying a single splitting time. We estimated the age of the split, all effective population sizes (including the ancestor), migration rates between the lake and each stream population (but not among stream populations) and the SNP mutation rate. The simulation was run in 80 replicates, each including 40 estimation loops with 100,000 coalescent simulations. To determine the best parameter estimates, we selected the 10 most likely replicate runs (that is, those with the smallest difference between the estimated and observed likelihood) and used this subset to calculate the mean for all parameters, along with their 95% confidence intervals (95 percentiles from bootstrap distributions based on 100,000 resamples). Because the lake population turned out to be particularly strongly influenced by selection, we explored an analogous model in which just the two stream populations most divergent from the lake (NID and GRA) split from an ancestor under gene flow. The joint SFS, the simulation template files, the parameter estimation files, and the command line settings used to run *fastsimcoal2.1* are provided as Supplementary Data 1–12.

To explore phylogenetic relationships among populations, we first reduced individual genotypes to single-letter code and eliminated individuals with >75% and SNPs with >15% missing data. We then used the R (ref. 57) package *phangorn* (ref. 58) to infer the most appropriate model of sequence evolution^59^ (‘GTR+G+I'). (The R language was used for all analytical procedures in this paper, unless noted otherwise.) Finally, we constructed unrooted maximum likelihood trees to infer the phylogeny of all four populations (based on 51,188 SNPs) and of the two lake samples only (55,561 SNPs). These analyses used no more than one SNP per RAD locus and required a MAF >0.2 across all populations (MAF >0.05 resulted in very similar results). Node support was assessed with 200 bootstrap replicates. The same data were also used to visualize genetic structure based on a principal coordinates analysis as implemented in the R package *ape* (ref. 60). Rooted phylogenies were constructed analogously by incorporating genotype data from geographically distant outgroup stickleback individuals, including the Pacific BEPA reference genome individual, at 14,429 SNPs ascertained in the populations from the Lake Constance basin.

### Genetic diversity

Two analyses were conducted to compare genetic diversity among the populations. For both, we only considered SNPs from our raw SNP matrix that occurred alone on a given RAD locus (that is, data from RAD loci harbouring multiple polymorphisms were ignored). Using only such ‘loner SNPs' avoided potential bias in the estimation of genetic diversity due to pseudo-SNPs caused by micro-indels. We further ignored those loner SNPs displaying a minor allele count <2 across all individuals pooled, thereby avoiding sequencing artifacts. We thus obtained a total of 62,332 genome-wide loner SNPs. As a first measure of diversity, we determined for each population the proportion of the total loner SNPs actually being polymorphic. To obtain a second diversity measure, we screened all loner SNPs for the presence of three alleles across all individuals pooled (‘tri-allelic loner SNPs'; the least frequent allele had to occur at least twice across all individuals). On average, one out of 169 loner SNPs proved tri-allelic (genome-wide total: 368). We then determined for each population the proportion of the total tri-allelic loner SNPs actually displaying all three alleles.

### Genome-wide LD

We quantified LD within each population using the squared correlation coefficient (*R*^2^) between pairs of SNPs. From the raw SNP matrix, we excluded SNPs that were tri-allelic or had >25% missing genotypes, and individuals with >75% missing diploid genotype calls. The remaining SNPs were filtered for two different MAF ranges (0.05–0.275 and 0.275–0.5). Only a single randomly chosen SNP was retained if multiple SNPs passed these thresholds for a pair of sister RAD loci (that is, the two RAD loci flanking the same restriction site). The final number of SNPs was 16,088 and 18,787 for the former and latter MAF range (marker number was adjusted to be equal for all populations). We then ran PLINK (ref. 61) with the command line '--ld-window 100 --ld-window-kb 100 --ld-window-r2 0' to calculate *R*^2^, enabling *R*^2^ values even below the default threshold of 0.2 to be reported. On average, this resulted in 142,249 *R*^2^ values for the 0.05–0.275 MAF range, and in 241,154 *R*^2^ values for the 0.275–0.5 MAF range. We then assigned the *R*^2^ values to 1-kb bins according to the physical distance between the two focal SNPs, and plotted the mean *R*^2^ for each bin from 1 to 100 kb. For the analysis of genome-wide LD decay with the full MAF range (0.05–0.5), we pooled the two MAF range specific PLINK outputs (one generated for the 0.05–0.275 and one for the 0.275–0.5 MAF range) before binning. Setting a MAF range of 0.05–0.5 right at the filtering step of the raw SNP matrix produced very similar results. To investigate more localized LD along chromosomes, we considered only *R*^2^ values between SNPs>2 kb but<50 kb apart (a range between 2 kb and 30 kb, or considering pairwise *R*^2^ values only produced similar results supporting identical conclusions). We determined the physical midpoints for all SNP pairs, binned the respective *R*^2^ values in non-overlapping 200-kb windows along the genome, and calculated average *R*^2^ for each window and population. Different window sizes (that is, 50 or 100 kb) yielded similar results supporting identical conclusions. To visualize localized differences in LD along the genome between the lake and GRA populations, we subtracted for each window the GRA *R*^2^ value from its lake counterpart, yielding a metric referred to as 'Delta *R*^2^'. We further calculated the correlation of *R*^2^ values between the lake and each stream population, using the above windows as data points. The magnitude of this correlation was evaluated against its empirical random distribution generated by permuting the *R*^2^ data over the windows 10,000 times.

### *F*_ST_-based identification of selected regions

Scans for genomic regions exhibiting strong differentiation were performed for each lake–stream combination. (We decided to refer to particularly high differentiation values as ‘extremes' rather than ‘outliers', as the outlier terminology implies a distinct class of loci.) Consistent with refs 5, 12, *F*_ST_ was calculated based on haplotype diversity. We considered only polymorphisms exhibiting a nucleotide coverage of at least 21 × in each population. To achieve adequate information to calculate genetic differentiation^62^, we further ignored SNPs with a MAF <0.2 across the focal lake and stream population pool. If multiple SNPs derived from the same RAD locus, we selected only the single one yielding the highest *F*_ST_ value (selecting instead based on maximum MAF, or at random, had no material influence on the results). Applying these stringent filters, we obtained 55,476, 57,119 and 60,052 genome-wide *F*_ST_ values for the BOH–lake, NID-lake and GRA-lake comparisons. To obtain regions suited for a detailed characterization of signatures of selection, we chose the 25 autosomal SNPs displaying the highest *F*_ST_ values across the three *F*_ST_ data sets combined (that is, 172,647 *F*_ST_ estimates from 79,770 unique SNPs). To ensure that each of these differentiation extremes represented an independent genomic region, SNPs were ignored if they were closer than 200 kb to a SNP already accepted as extreme.

### Haplotype-based identification of selected regions

Our *F*_ST_-based search for evidence of positive selection was complemented with haplotype-based statistics proving particularly powerful to detect incomplete selective sweeps^32^,^33^. However, they rely on relatively high marker resolution and robust sequence coverage in many individuals; requirements met by our study (see above and Supplementary Table 2). From the raw SNP matrix, we first excluded SNPs that were tri-allelic, had >40% missing genotypes, or did not reach a MAF of 0.05. We further excluded individuals with >75% missing diploid genotype calls after SNP-filtering. *fastPHASE* (ref. 63) was then used to reconstruct haplotypes and missing genotypes separately for each chromosome. We classified individuals according to their population (-u option) and increased the number of iterations of the EM algorithm to 50 (-C option; default is 25) and the number of sampled haplotypes to 100 (-H option; default is 20). *fastPHASE* output files were then imported into the R package *rehh* (ref. 64) to obtain the following haplotype-based statistics: EHH^65^ (allele-specific ‘Extended Haplotype Homozygosity'), EHHS^65^ (population-specific weighted average of EHH across both alleles), iHH^66^ ('integrated Haplotype Homozygosity'), iHS^66^ (‘integrated Haplotype Score') and Rsb^34^ (the standardized ratio of integrated EHHS from two populations). iHS was calculated separately for each of the four populations using the 'scan_hh' and 'ihh2ihs' commands ('minmaf', the MAF threshold, was set to 0.05; '-freqbin' was set to 0, but setting this option to 0.05 or 0.1 resulted in qualitatively similar results supporting identical conclusions). Rsb was calculated for each of the three possible lake–stream comparisons by applying default parameters ('ies2rsb' command). We obtained a total of 87,738 Rsb values (corresponding to an average marker distance of 4.8 kb), which were screened for extremes (that is, values below −4 or above 4 (refs 34, 35)). Haplotype decay around *F*_ST_ and Rsb extremes was calculated and visualized using the 'calc_ehh' and the 'calc_ehhs' option at default.

### Analyses specific to lateral plating

To screen the genome for loci influencing the lateral plate phenotype, we performed a BSA by assigning 24 completely plated stream individuals to one phenotypic group, and 24 low-plated stream individuals to another group (lateral plate phenotyping followed ref. 23 and is presented in Supplementary Table 2). This assignment considered all three stream populations but ignored the (mostly completely plated) lake fish, thus avoiding confounding signals of lake–stream divergence (that is, signals unrelated to plate phenotype). Based on 61,822 SNPs, we then carried out a genome-wide *F*_ST_ scan by treating the phenotypic groups as populations, but otherwise following all conventions described above for the population-based *F*_ST_ calculations.

To examine if *Eda* was recognized as a selected locus in a standard *F*_ST_ scan, we applied *BayeScan*^40^ to the SNP data set from the GRA-lake comparison (60,052 markers), that is, the population pair with the strongest differentiation at *Eda*. *BayeScan* was run with default settings except that we used 300 as prior odds for neutrality—according to the software manual an appropriate value for this data set. However, a second analysis was performed with the default prior odds of 10, which is expected to produce more liberal results.

For the *Eda*-specific analyses of haplotype structure, we created three pools: a first pool with all completely plated stream individuals, a second pool with all low-plated stream individuals (both *N*=24), and a third pool with all completely plated lake individuals (*N*=19). We calculated and plotted EHHS for each pool around the SNP exhibiting the highest *F*_ST_ value in the above bulk segregant genome scan (bp-position 12,832,658 on chromosome IV). Finally, we subtracted iHS values from the completely plated stream individuals from the corresponding values in the low-plated stream individuals ('Delta iHS') across chromosome IV (*N*=5,626; average marker distance=6 kb). Delta iHS was then averaged and plotted in non-overlapping 100-kb windows (different window sizes led to identical conclusions).

### Identification and characterization of inversions

Our approach to detecting inversions was based on the expectation that the two inversion types (collinear and inverted), representing two isolated populations, differ in their magnitude of divergence from the reference genome. This should cause differential read alignment success across inverted genomic regions. Inversions should thus be revealed by a physically extended distortion of the relative RAD locus sequence coverage between two populations if these populations differ in the frequency of the inversion types (Supplementary Fig. 11). The same logic recently enabled the identification of evolutionary strata on the stickleback sex chromosome^42^. We therefore screened all 372,884 RAD loci for population-specific sequence coverage, excluding those with a total sequence coverage below 200 across all populations and those located in genomic regions unanchored to chromosomes, thus obtaining 290,170 informative loci. For each stream population, we calculated the RAD locus-specific stream to lake coverage ratio. Next, we divided the chromosomes in non-overlapping 20-kb windows (21,048 in total) and calculated the average coverage ratio among the RAD loci for each one of them (using the coverage variance among RAD loci within windows produced very similar results). The median number of RAD loci per window was 13. Finally, we looked for distortions in the coverage ratio extending over multiple adjacent windows, suggesting the presence of an inversion. We note that this analysis based on read coverage was limited to the detection of relatively large inversions exhibiting substantial sequence divergence.

To confirm that the above sequence coverage method reliably detects inversions, we used RAD loci near an expected inversion breakpoint in two of the three emerging candidate regions to design PCR primer pairs across the breakpoint boundaries. These primer pairs were expected to yield a PCR product only for the inversion type occurring in the streams. Ten to 13 individuals representing a given inversion type were subjected to long-range PCR and inspected for the presence or absence of amplification (further details are given in Supplementary Fig. 12).

Next, we examined allelic diversity and minor allele frequencies (MAFs) around the three detected inversions. For this, we screened each of the four population samples separately for polymorphisms with >50% available genotype calls (singletons were omitted to exclude technical artifacts) and calculated haplotype diversity (that is, an analogue of heterozygosity ranging from 0 to 0.5) and the MAF at each SNP. RAD loci were allowed to contribute a single SNP only, keeping the one with the highest diversity when multiple SNPs occurred on the same locus (drawing a SNP at random or averaging diversity estimates of multiple SNPs per RAD locus yielded very similar results). Diversity was visualized using R's implementation of LOESS (locally weighted scatterplot smoothing; LOESS was used for all smoothing in this paper). The MAF frequency distribution within the inversions was plotted for the lake and for the stream population displaying the strongest inversion frequency shift from the lake. For this population, we also plotted the genome-wide MAF distribution.

To investigate LD patterns around the three inversions and to refine their physical boundaries, we calculated LD as the correlation among unphased SNP alleles using the *R*^2^ statistic implemented in *mcld* (ref. 67). Only bi-allelic SNPs with <25% missing data and individuals with<50% missing *diploid* genotype calls were considered. When multiple SNPs were located on sister RAD loci, only a single randomly picked SNP was retained. For the calculation of LD, we applied different MAF filters, including a 0.15 MAF range centred on the MAF peak reflecting the relative frequency of the two variants at each inversion (see MAF analysis above). Patterns of LD around the inversions were visualized using the *LDheatmap* (ref. 68) R package for the stream population displaying the strongest inversion frequency shift from the lake (analysing the other stream populations yielded very similar estimates of the inversion breakpoint positions).

To construct haplotype genealogies for the inversions using individuals from the Lake Constance basin only, we first extracted the SNPs in each inversion, (SNPs closer than 20 kb to the inversion breakpoints identified in the LD analysis above were not considered). Next, we excluded SNPs with a MAF<0.05 and with >25% missing genotypes. Different MAF ranges (that is, 0.1–0.5 or 0.2–0.4) led to identical conclusions. Individuals with >75% missing *diploid* genotypes after removing low-quality SNPs were excluded. When multiple SNPs per sister RAD loci passed the above filters, we only retained the one with the highest MAF (choosing a random SNP yielded similar results). For the largest inversion (located on ChrXXI), we randomly subsampled the resulting SNP panel to a total of 173 SNPs to reduce complexity. Haplotype reconstruction used *PHASE 2.1* (ref. 69), optimized by specifying the physical position of all polymorphisms and increasing the number of search iterations to 499. Five independent runs were performed with different seeds to confirm consistency among the results. Haplotype alignments were used to infer phylogenetic trees with *RAxML* v.8.0.0 (ref. 70), using the GTRCAT model of sequence evolution with rate heterogeneity among sites. Based on sequence alignments and phylogenetic trees, we constructed and visualized haplotype genealogies with *Fitchi* (Matschiner, M.: Fitchi: Haplotype genealogy graphs based on Fitch distances. http://www.evoinformatics.eu/fitchi, 2015), using a minimal node size of two haplotypes for display (-n option). To construct haplotype networks including individuals from across the stickleback's geographic range, we randomly selected 20 SNPs from the Lake Constance-specific haplotype genealogies, and inferred the genotypes at these SNPs for a total of 11 freshwater and 10 marine stickleback specimens^11^ based on the ENSEMBL and the UCSC Stickleback Genome Browsers. The resulting SNPs (12, 13 and 14 for the ChrI, ChrXI and ChrXXI inversions) were used for haplotype network construction and visualization as described above.

The Lake Constance-specific haplotype networks allowed us to unambiguously infer diploid genotypes at all three inversions for our main study individuals. Of these individuals, 33 had already been RAD sequenced previously using the Sbf1 restriction enzyme^62^, allowing us to determine SNPs on Sbf1 RAD loci diagnostic for the two variants at each inversion. At these diagnostic SNPs, we then determined the diploid genotypes in 27 lake and 27 stream stickleback from the Lake Geneva basin^21^. For the stream individuals, Sbf1 RAD data were already available^38^. For the Lake Geneva individuals, however, RAD sequence data were generated specifically for this study, following the protocol described in ref. 5. The SNP data from all individuals from the Lake Geneva basin were then used to search for the presence of inversion polymorphisms in this lake–stream system, to determine the frequencies of the inversion types in each population, and additionally to conduct an *F*_ST_-based lake–stream genome scan.

To explore the short-term recombination rate at the inversions, we inspected genotype data from an F2 laboratory intercross^42^. This revealed that the two parental stickleback individuals used to initiate the cross (a male from Lake Constance and a female from a tributary stream of Lake Geneva) were fixed for different inversion types at the ChrI inversion (but not at the two other inversions). We therefore counted crossovers between SNPs across the ChrI inversion region in all 282 F2 individuals. As a negative control, we did the same around the ChrXI and ChrXXI inversions. To address the theoretical prediction that large inversions should maintain some genetic exchange due to double crossovers (gene conversion is considered less important)^47^, we assigned stream individuals from the Lake Constance basin homozygous for one or the other inversion type at the ChrI inversion to separate groups (*N*=15 and 20 for the stream and lake inversion type, defined according to Fig. 7c). These groups were then used to perform an *F*_ST_-based differentiation scan. Additionally, using the same groups, we determined the number and location of loner SNPs specific to each inversion type, or shared between the types, within and around the ChrI inversion. Analogous analyses for the ChrXI and ChrXXI inversions were not possible because here individuals homozygous for the stream inversion type were too rare (Fig. 7c).

## Additional information

**Accession codes:** RAD-seq data generated for this study have been deposited in the NCBI Sequence Read Archive under BioProject number PRJNA273792 (Nsi1 data from the Lake Constance region) and PRJNA284945 (Sbf1 data from the Lake Geneva region).

**How to cite this article:** Roesti, M. *et al*. The genomics of ecological vicariance in threespine stickleback fish. *Nat. Commun.* 6:8767 doi: 10.1038/ncomms9767 (2015).

## Supplementary Material

Supplementary Information Supplementary Figures 1-15, Supplementary Tables 1-2 and Supplementary References

Supplementary Data 1 The observed BOH-Lake joint site frequency spectrum (SFS) input file for the 'full model' including all study populations

Supplementary Data 2 The observed NID-Lake joint site frequency spectrum (SFS) input file for the 'full model' including all study populations

Supplementary Data 3 The observed NID-BOH joint site frequency spectrum (SFS) input file for the 'full model' including all study populations

Supplementary Data 4 The observed GRA-Lake joint site frequency spectrum (SFS) input file for the 'full model' including all study populations

Supplementary Data 5 The observed GRA-BOH joint site frequency spectrum (SFS) input file for the 'full model' including all study populations

Supplementary Data 6 The observed GRA-NID joint site frequency spectrum (SFS) input file for the 'full model' including all study populations

Supplementary Data 7 Fastsimcoal parameter estimation file for the 'full model' including all study populations

Supplementary Data 8 Fastsimcoal simulation template file for the 'full model' including all study populations

Supplementary Data 9 The observed GRA-NID joint site frequency spectrum (SFS) input file for the 'GRA_NID model' including only the GRA and NID populations

Supplementary Data 10 Fastsimcoal parameter estimation file for the 'GRA_NID model' including only the GRA and NID populations

Supplementary Data 11 Fastsimcoal simulation template file for the 'GRA_NID model' including only the GRA and NID populations

Supplementary Data 12 README file for the fastsimcoal analyses, including command lines and details on the input files
