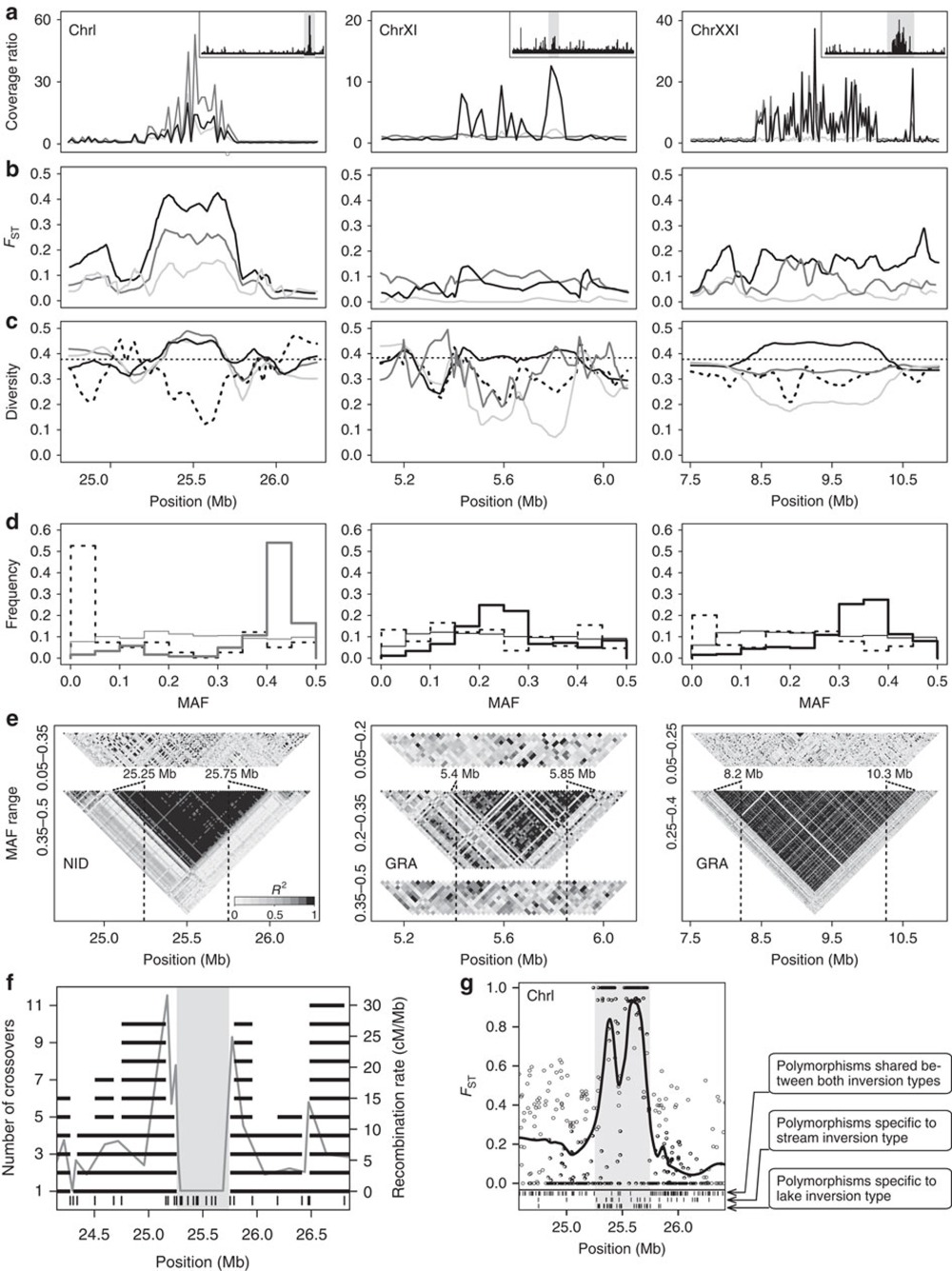# Erratum: The genomics of ecological vicariance in threespine stickleback fish

**DOI:** 10.1038/ncomms10229

**Published:** 2015-12-15

**Authors:** Marius Roesti, Benjamin Kueng, Dario Moser, Daniel Berner

*Nature Communications* 6: Article Number: 876710.1038/ncomms9767 (2015); Published 11102015; Updated 12152015

The recombination rate around the chromosomal inversion in Fig. 6f of this Article was inadvertently omitted during the production process. The correct version of Fig. 6f appears below.

**Figure 6**