# Supplementary material for: The genomics of ecological vicariance in threespine stickleback fish
Source: Nat Commun. 2015 Nov 10;6:8767. doi: 10.1038/ncomms9767 (PMC4659939; doi:10.1038/ncomms9767)
Supplement: Supplementary Information — Supplementary Figures 1-15, Supplementary Tables 1-2 and Supplementary References [file ncomms9767-s1.pdf]

SUPPLEMENTARY FIGURES

Supplementary Figure 1

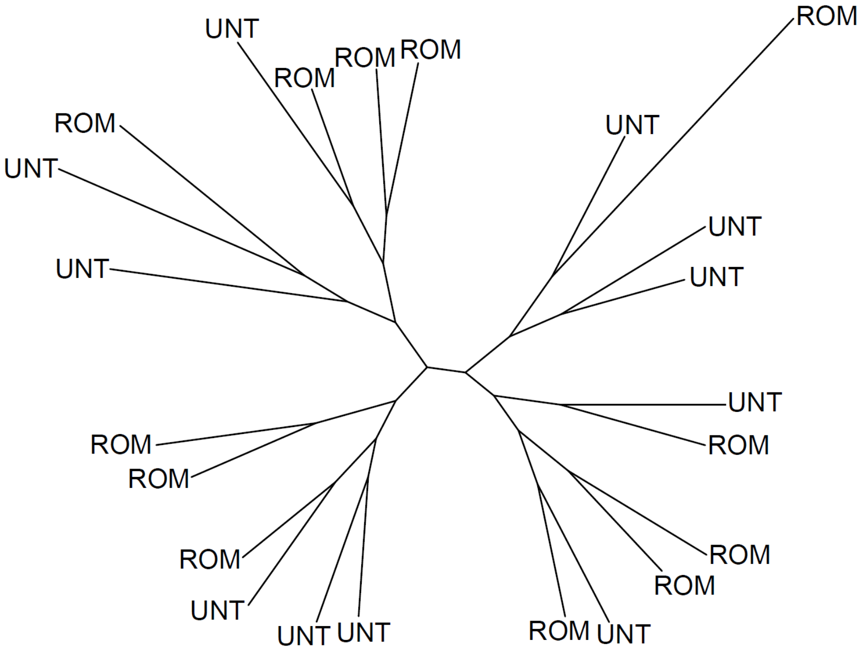

Supplementary Figure 1. Phylogenetic analysis restricted to the two stickleback samples from Lake Constance. The unrooted maximum likelihood tree is based on 55,561 genome-wide SNPs in fish sampled from two lake sites approximately 20 km apart. The sites are Romanshorn (ROM), Switzerland, western lake shore, and Unteruhldingen (UNT), Germany, eastern shore (for geographic details see ref. 1). Consistent with a genome-wide median F_{ST} of zero between ROM and UNT, the phylogeny reveals the absence of genetic structure between the two sites, indicating that Lake Constance is inhabited by a single panmictic stickleback population. The same conclusion was drawn earlier based on microsatellite markers and stickleback samples from four different lake sites[1].

Supplementary Figure 2

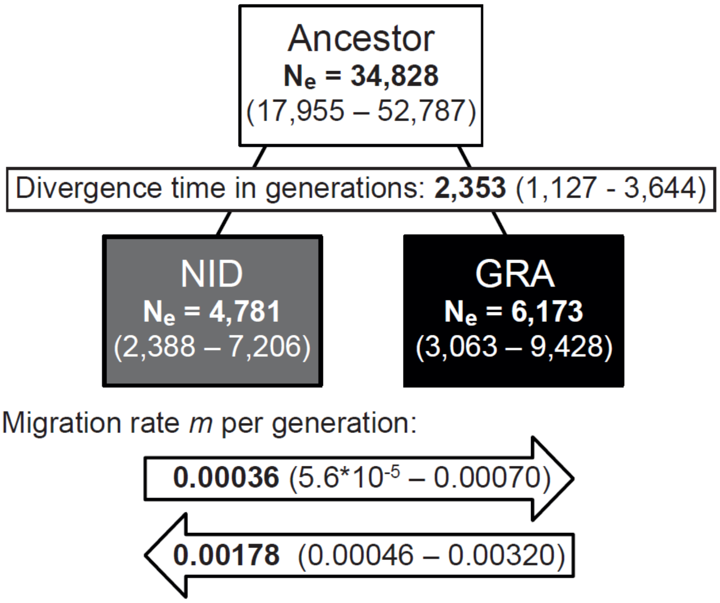

Supplementary Figure 2. Demographic analysis based on a reduced model including the GRA and NID stream populations only. Plotting conventions are as in the full model including all study populations (Fig. 1b). The GRA and NID populations are the genetically most variable of our study populations (see main text). In the reduced model, the split between GRA and NID from a common ancestor is estimated to have occurred more recently compared to the full model, although the confidence intervals overlap widely between the models. A potential reason for the deeper splitting time in the full model is upward bias due to extensive genome-wide selective sweeps experienced by the lake population. We thus consider the splitting time estimate from the reduced model a better approximation of the true time since stickleback colonized the Lake Constance basin. However, both models support qualitatively similar conclusions about the colonization history of the Lake Constance basin.

Supplementary Figure 3

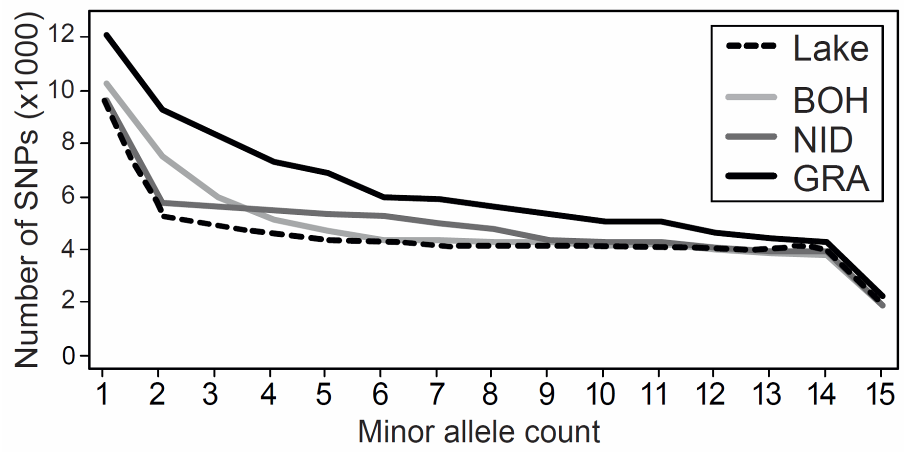

Supplementary Figure 3. Observed site frequency spectra (SFS) of the four study populations. The X-axis indicates the occurrence of the minor allele among 30 randomly sampled nucleotides at a given genome position (the minor allele *frequency* (MAF) would thus be obtained by dividing the counts by 30). The Y-axis gives the number of sites falling into each minor allele count class in each population. Like the joint SFS used for demographic inference, these population-specific SFS are based on 14.8 million nucleotide positions, although for the ease of presentation, only the polymorphic sites (i.e., minor allele count > 0) are shown. Note the low number of polymorphisms across most minor allele count classes in the lake population relative to the stream populations (especially GRA). Accordingly, the lake population exhibited the highest proportion of monomorphic sites (minor allele count = 0); in millions, lake: 14.770; BOH: 14.765; NID: 14.764; GRA: 14.745.

Supplementary Figure 4

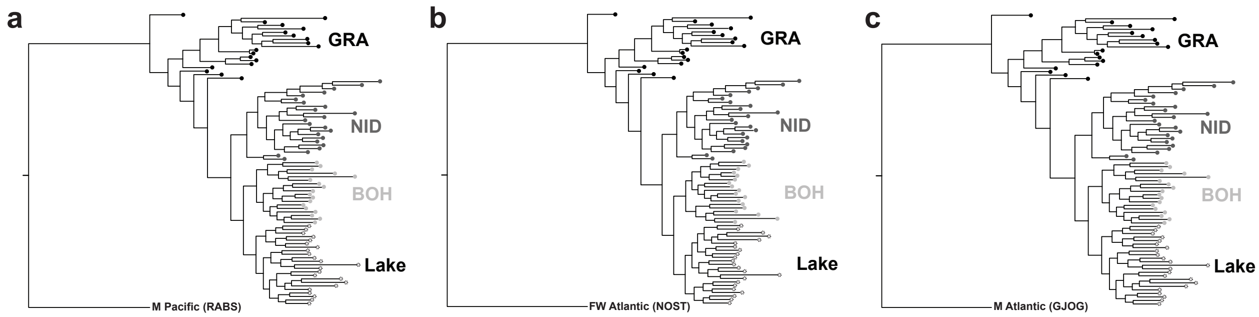

Supplementary Figure 4. Phylogenetic relationships among the Lake Constance study populations rooted using different outgroups. To verify the robustness of the rooted ML phylogeny by using the reference genome individual (a freshwater individual from the Pacific, see Fig. 1c) as an outgroup, we generated additional trees using several other outgroups, including **(a)** a marine Pacific (sampling population: 'Rabbit Slough', Alaska), **(b)** an freshwater Atlantic (sampling population: 'Norway Stream', Norway), and **(c)** a marine Atlantic (sampling population: 'Gjögur', Iceland) stickleback individual. Genotypes for these individuals were retrieved from the 'Stickleback Genome Browser' (http://sticklebrowser.stanford.edu/)[2]. These analyses consistently resulted in very similar tree topologies supporting identical conclusions.

Supplementary Figure 5

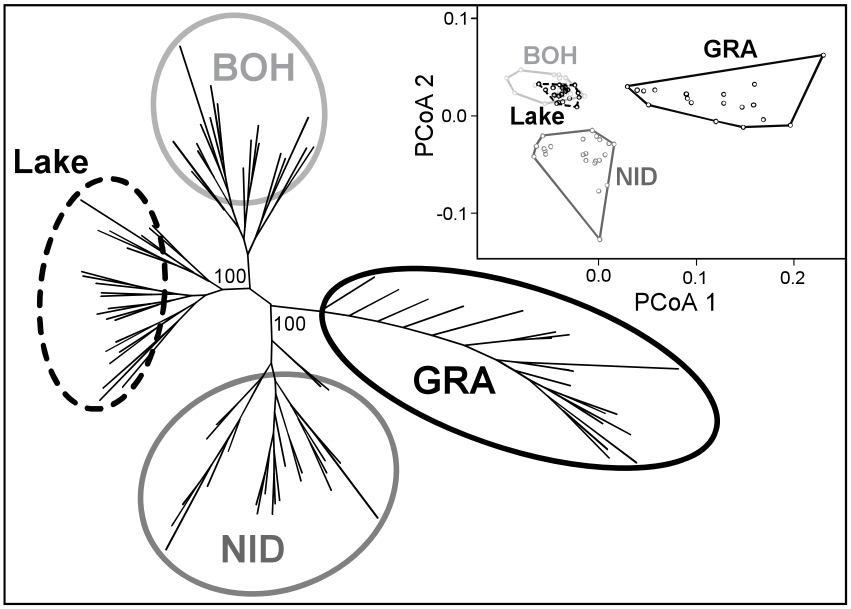

Supplementary Figure 5. Phylogenetic and principal coordinate analysis of the four stickleback populations from the Lake Constance basin. The unrooted maximum likelihood tree (based on 51,188 SNPs; bootstrap support in percent is given for the key nodes) reveals reciprocal monophyly of the four populations. Both the tree and the principal coordinate ordination (insert) further show the close relatedness of the lake and the BOH population, and that genetic diversity increases from the lake population to the BOH, NID, and GRA stream populations.

Supplementary Figure 6

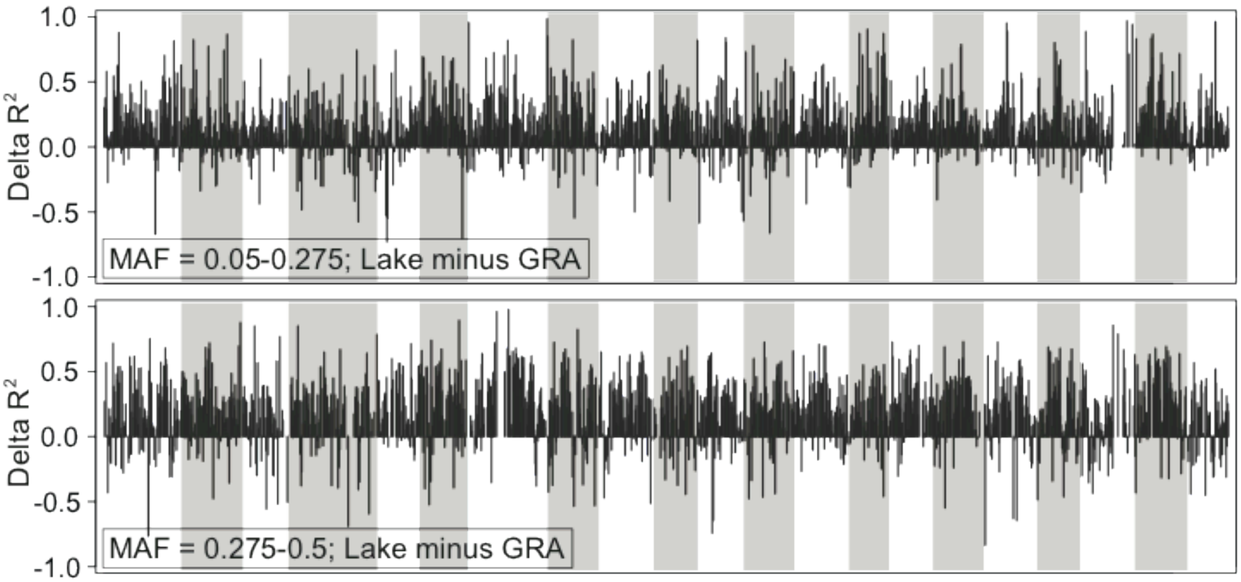

Supplementary Figure 6. Influence of using SNPs from different MAF classes on the difference in LD between the lake and the GRA population. Shown is Delta R^2 (see Fig. 2b) based on low-MAF (top) and high-MAF (bottom) SNPs. The MAF classes are separated using the same thresholds as used in the insert of Fig. 2a. Irrespective of the MAF class, LD is higher in the lake than in GRA along most of the genome.

Supplementary Figure 7

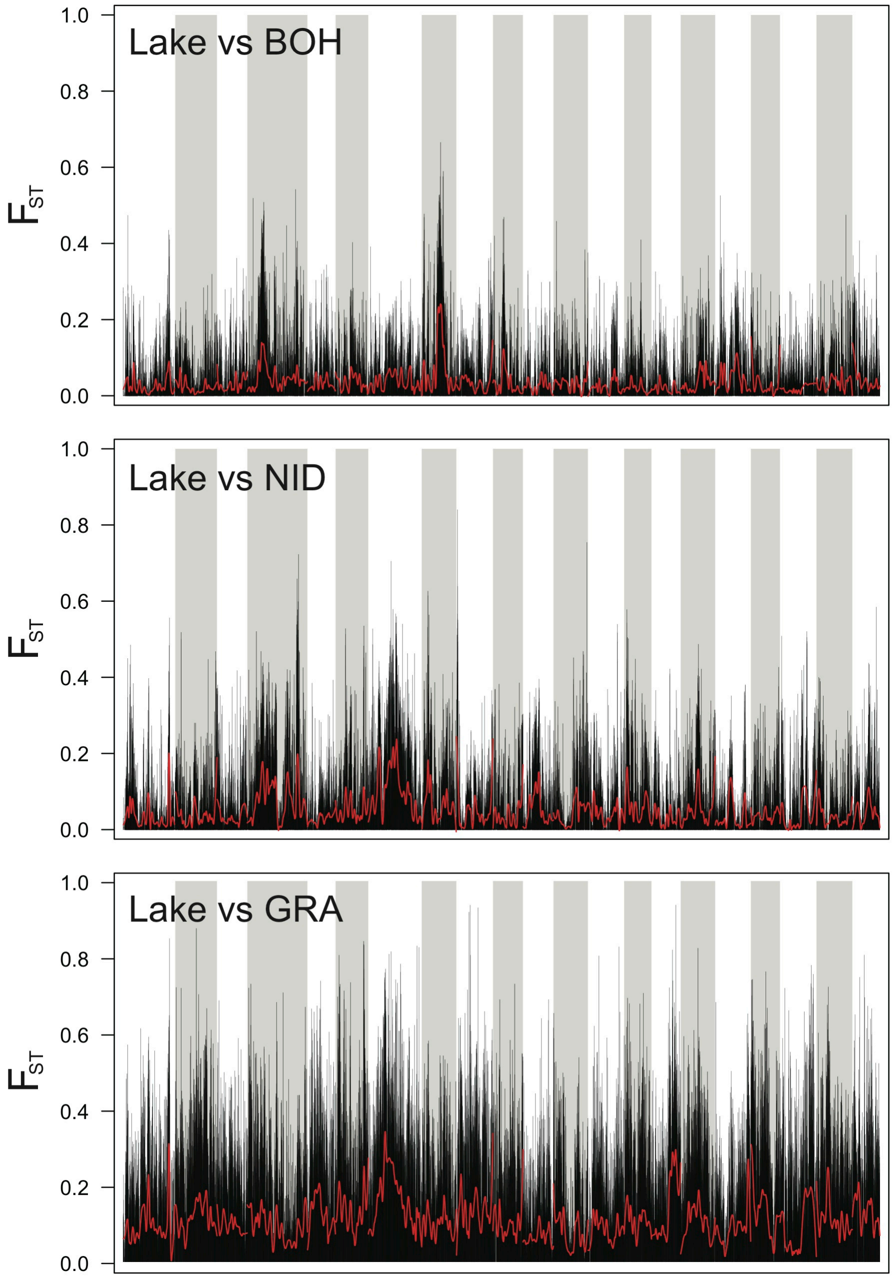

Supplementary Figure 7. Genome-wide divergence (F_{ST}) for all lake-stream comparisons. The black vertical lines represent the raw F_{ST} values, the red profiles show these values smoothed by LOESS, and the background shading separates the 21 chromosomes. Note the increase in baseline differentiation from BOH (median F_{ST} = 0.005; 55,476 SNPs) to NID (0.013; 57,119 SNPs) and GRA (0.061; 60,052 SNPs).

Supplementary Figure 8

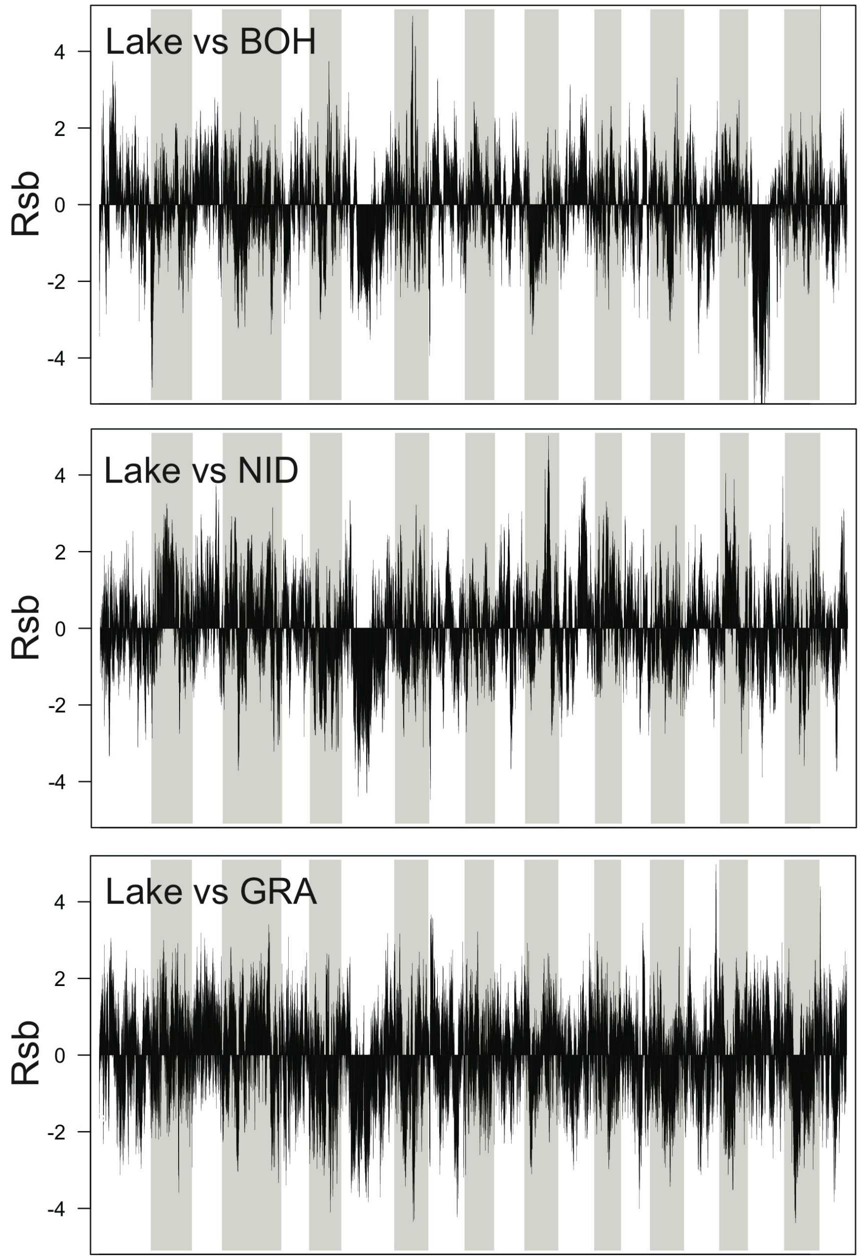

Supplementary Figure 8. Difference in haplotype decay around genome-wide SNPs, as captured by Rsb, for each lake-stream population pairing. The background shading separates the 21 chromosomes. A total of 87,738 SNPs were used in all lake-stream comparisons.

Supplementary Figure 9

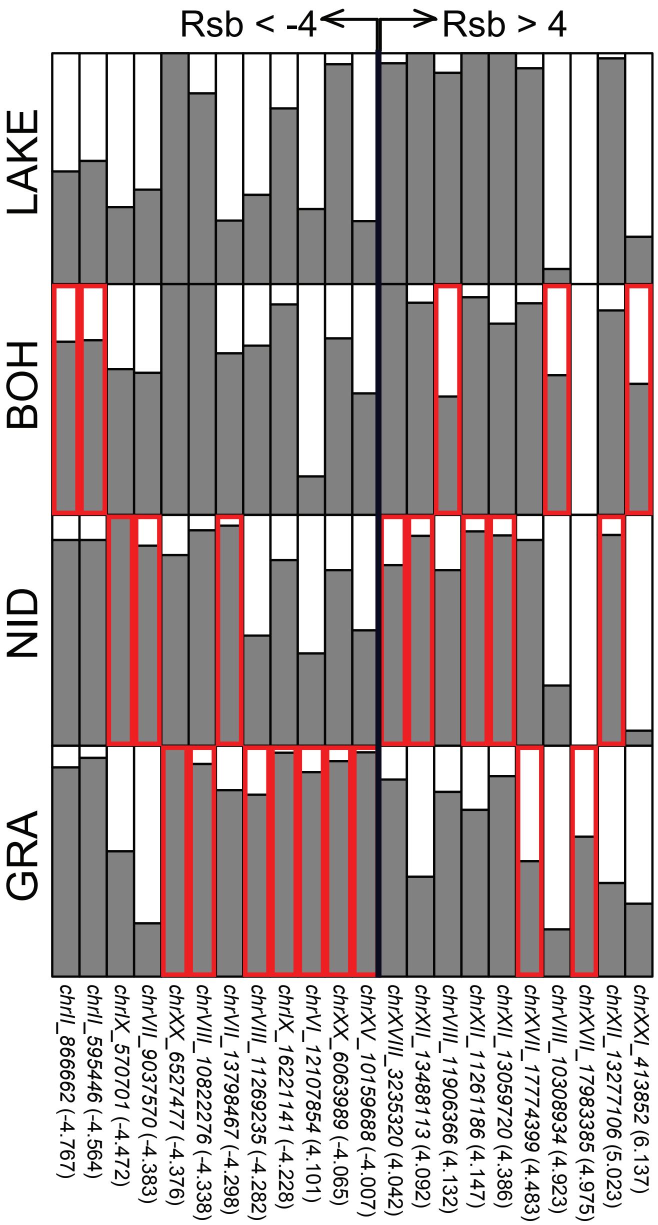

Supplementary Figure 9. Allele frequencies within each population at the top 22 lake-stream Rsb extremes. At each Rsb extreme (columns), the stream population producing an absolute Rsb > 4 in comparison to the lake is framed in red. On the bottom, the genomic position and the highest Rsb value observed across all lake-stream comparisons are given for each Rsb extreme. *Negative* Rsb extremes generally display relatively balanced polymorphism in the lake, but strong bias toward a specific allele in the stream(s), hence suggesting stream-specific selective sweeps. By contrast, *positive* Rsb extremes tend to exhibit relatively balanced polymorphism in the streams but are near fixation for a specific allele in the lake, thus indicating lake-specific selective sweeps.

Supplementary Figure 10

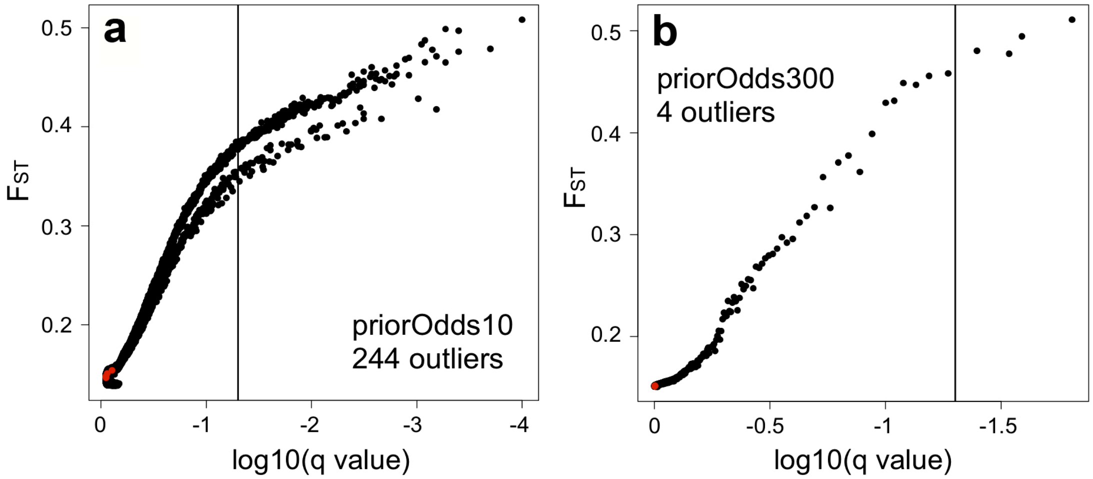

Supplementary Figure 10. BayeScan divergence outlier analysis in the Lake Constance and GRA stream population pair. Analysis to explore if markers near the Ectodysplasin (*Eda*) gene, known to be under divergent selection between these populations, are recognized as selection outliers by a popular outlier detection program not requiring a reference genome. The analysis used 60,052 SNPs, and was run both with default settings **(a)**, and with the prior odds for neutrality increased to 300 **(b)** (default is 10). According to the software manual, the latter setting should be more appropriate for our large marker data set, while the default is perhaps too liberal. The graphics display the results of these two outlier scans, with the five markers near *Eda* exhibiting the highest F_{ST} in our differentiation scan printed in red (see top panel in Fig. 5c; positions on ChrIV: 12,815,791; 12,818,350; 12,818,237; 12,820,744; 12,822,878). SNPs on the right of the vertical line (244 and 4 in the two scans) qualify as differentiation outliers at a false discovery rate of 0.05. None of the markers near *Eda* are identified as outliers by BayeScan.

Supplementary Figure 11

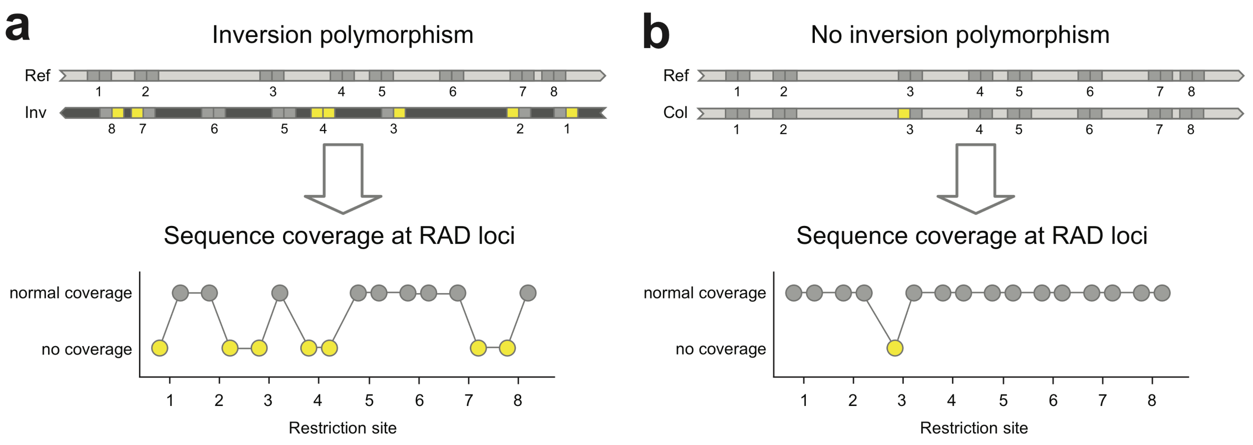

Supplementary Figure 11. Strategy for the detection of inversion polymorphisms using RAD locus coverage. (a) An individual harboring the inverted inversion type ('Inv'; dark gray background shading) relative to the reference sequence ('Ref'; light gray background) (for simplicity, individuals are haploid in this figure). The small squares represent the two RAD loci flanking restriction enzyme cutting sites to either side (sister RAD loci). If the 'Inv' inversion type shows substantial divergence from the reference, individuals carrying this type will lack sequence coverage at many RAD loci when aligned to the reference (RAD loci too strongly differentiated to align to the reference are shown as yellow squares). The bottom panel shows the resulting pattern of sequence coverage across RAD loci for this inversion type. **(b)** An individual carrying the inversion type collinear ('Col') to the reference (top), and the resulting sequence coverage along this chromosomal segment (bottom).

If the different inversion types segregate at different frequencies within two populations, mean sequence coverage across chromosome windows within the inversion will be biased toward the population in which the 'Inv' type is less common, relative to chromosome segments outside the inversion. An analogous signature emerges when comparing the variance in sequence coverage across chromosome windows within and outside inversions between populations. Both signals, i.e., bias in the ratio of mean sequence coverage and coverage variance between populations along the genome, were exploited in our study and both consistently detected the three inversions, although only the former is presented (Fig. 6a). (Note that distortions in mean coverage and coverage variance along chromosomes can also be used to detect inversions in a single population, although the comparison of populations provides additional information on shifts in inversion frequencies.)

The prerequisites for the above inversion detection approaches are that the inverted and collinear segments display substantial sequence divergence (recent inversions cannot be detected), and that the density of restriction sites is high enough to allow calculating the bias in the ratio of mean sequence coverage or the coverage variance between populations in relatively small chromosome windows while still integrating coverage data from a reasonably large number of RAD loci (a low-frequency restriction enzyme digest will allow detecting large inversions only). Moreover, comparing coverage statistics between populations will detect inversion only when these populations have diverged sufficiently in the frequency of the inversion types.

Supplementary Figure 12

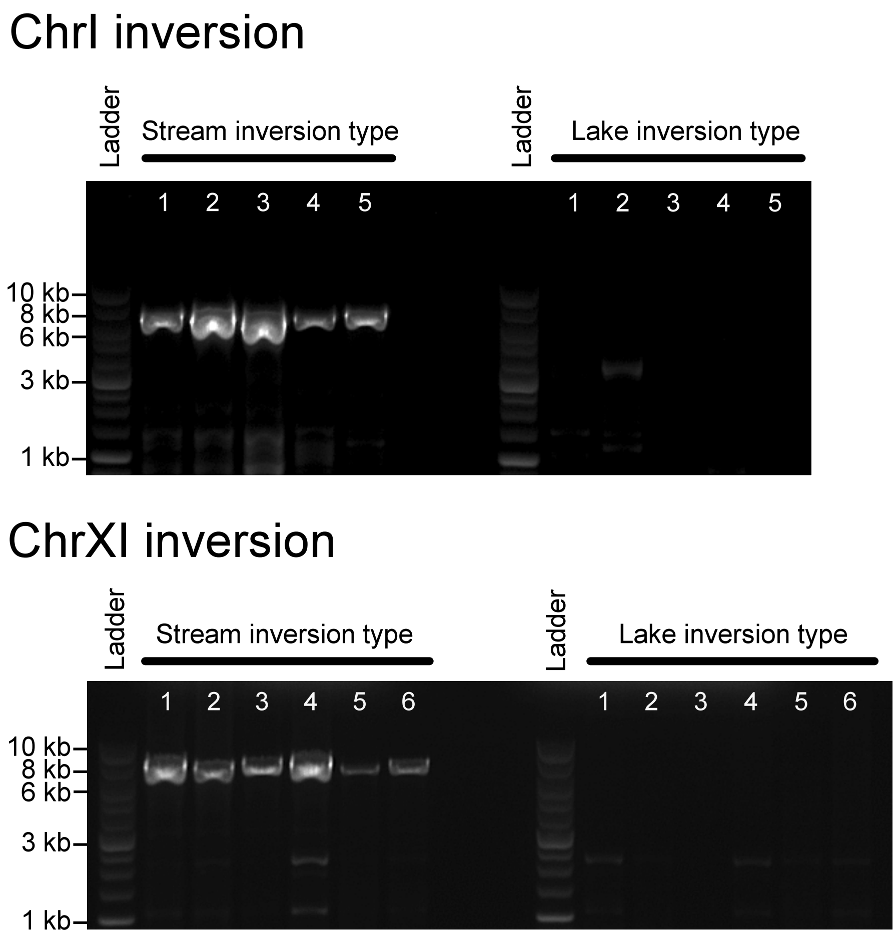

Supplementary Figure 12. Confirming inversions by inversion type-specific PCR across expected breakpoints. For the putative ChrI and ChrXI inversions detected based on RAD sequence coverage, we used RAD loci flanking one inversion breakpoint to design PCR primer pairs expected to yield a PCR product for the inversion type specific to the streams, but no product for the inversion type fixed in the lake (see Fig. 7c). The underlying RAD loci were required to display robust alignment to the reference genome in *all* populations, thus ensuring that any absence of PCR amplification was due to the physical relocation of a primer site, and not to the degeneration of a primer site. For the ChrI inversion, we assessed 13 individuals homozygous for the stream type, of which nine (70%) amplified successfully, and ten individuals homozygous for the lake type, of which none amplified (five individuals of each group are shown on the gel image). For the ChrXI inversion, we assessed five individuals homozygous for the stream type and seven heterozygous

individuals, all of which amplified successfully. By contrast, none of the ten individuals homozygous for the lake type amplified (six individuals of each group are visualized; the individuals 5 and 6 in the stream inversion group are heterozygous). These analyses thus confirm that the candidate regions are truly inversions. Note that the ChrI (and also the ChrXXI) inversion has been confirmed independently through PCR, using different primer pairs than in the present study[2]. The ChrXI inversion, however, has not previously been verified by PCR.

As representatives of both inversion types, our PCRs considered primarily individuals from the stream populations in the Lake Constance basin (these populations are polymorphic for the inversions; Fig. 7). A few individuals from the Lake Geneva basin (Fig. 7c), however, were included in all reactions, which showed that geographic origin did not influence amplification success. The primer combinations used for this analysis were 5'- GCTGGTCAATATGTCCACTC-'3 (forward) and 5'- GTTACAATATGCCAATTACATGTC-'3 (reverse) for ChrI (approximate expected product size: 6.2 kb), and 5'-GGAGAAGCCTCAACCTATACG-'3 (forward) and 5'-GGTGAGCAACTTGAACCAAG-'3 (reverse) for ChrXI (6.8 kb). Long-range PCRs were performed with 37 cycles using Phusion High-Fidelity PCR chemistry (New England BioLabs), following the manufacturer's protocol.

Supplementary Figure 13

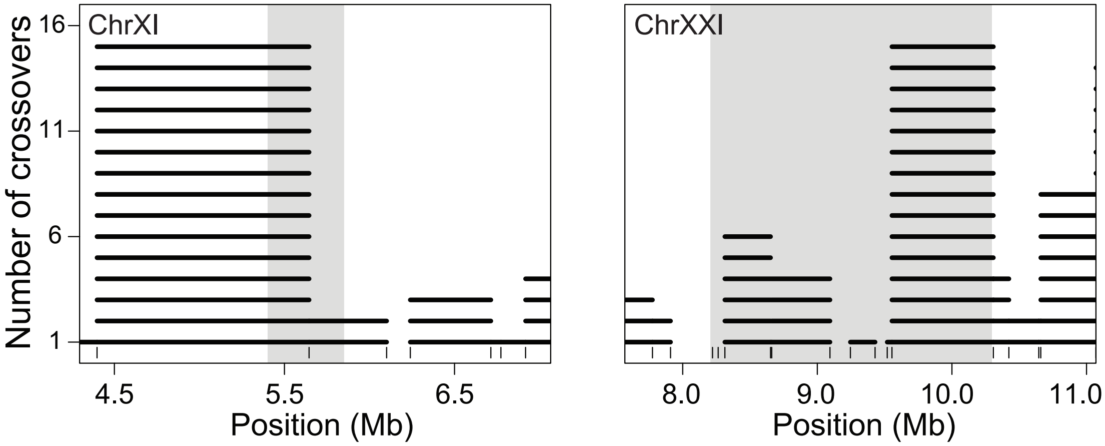

Supplementary Figure 13. Recombination rate around the ChrXI and ChrXXI inversions in a laboratory cross population. Plotting conventions are as in Fig. 6f. For the ChrXI and ChrXXI inversion, the cross population underlying the recombination analysis reported in Fig. 6f is monomorphic. We here show that, as expected, recombination in these regions is *not* suppressed, thus providing a negative control for the analysis presented in Fig. 6f.

Supplementary Figure 14

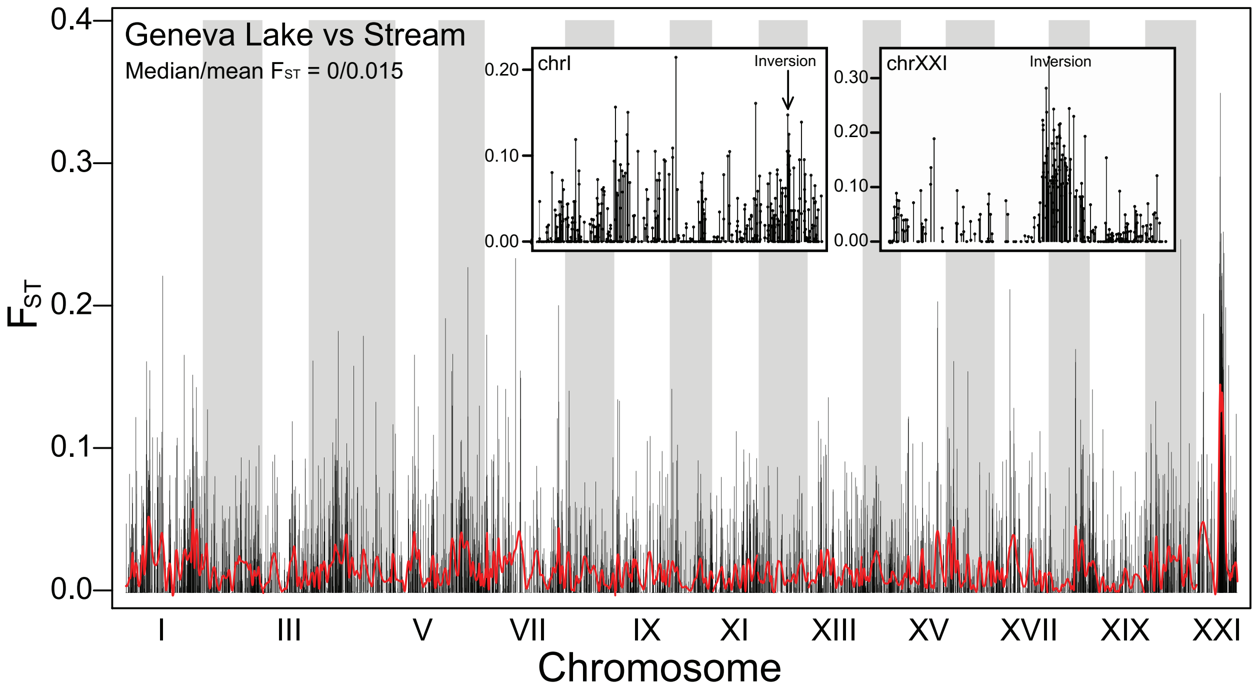

Supplementary Figure 14. Genetic differentiation (F_{ST}) between the Lake Geneva population, and a stream population from a tributary of this lake. The black vertical lines represent the raw lake-stream F_{ST} values, the red profiles show these values smoothed by LOESS, and the background shading separates the 21 chromosomes. The genome region displaying the strongest differentiation is located on ChrXXI and coincides with the large inversion on that chromosome (right insert; average F_{ST} across this inversion: 0.160). Relative to the low genome-wide baseline differentiation (given in top-left corner), the ChrI inversion also exhibits strong lake-stream divergence (left insert; average F_{ST} across this inversion: 0.084).

Supplementary Figure 15

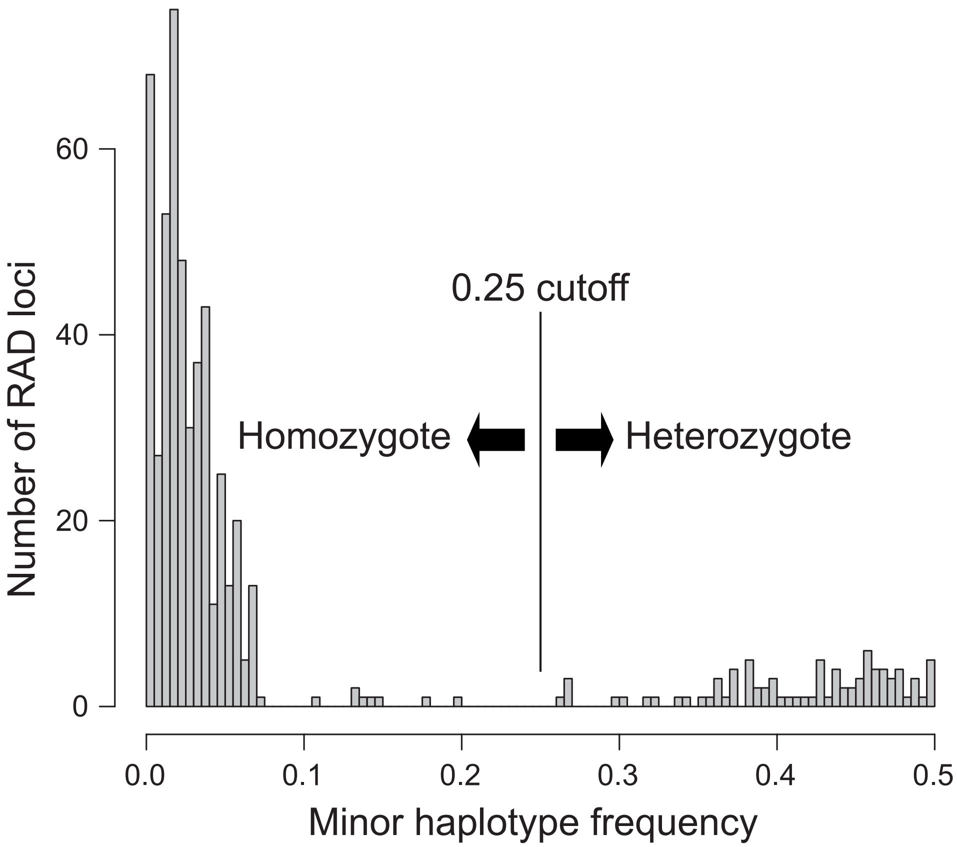

Supplementary Figure 15. Determining an appropriate threshold for distinguishing homozygote and heterozygote RAD loci during consensus genotyping. To identify this threshold, we determined the frequency of all haplotypes occurring at 250 haphazardly chosen RAD loci in each of three individuals displaying low, medium, and high raw Illumina sequence coverage. Among these 750 total RAD loci, we discarded those in which the two most frequent haplotypes together failed to account for > 70% of all haplotypes and/or to reach a sum of 15 (see Methods). Across the remaining 562 RAD loci, we then calculated the minor haplotype frequency, defined as the count of the second most frequent haplotype divided by the sum of the two most frequent haplotypes. The distribution of this statistic indicated that a cutoff around 0.25 effectively separated truly heterozygous RAD loci from those appearing variable because of a technical artifact.

SUPPLEMENTARY TABLES

Supplementary Table 1. Genetic diversity within each of the four study populations. Diversity is calculated based exclusively on 'loner SNPs' (i.e., SNP occurring alone on their RAD locus, see Methods). The first two data columns indicate the number and corresponding proportion of the total loner SNPs (N = 62,332) actually being polymorphic within each population (in parentheses the proportions are scaled such that the lake is 100%). This proportion is lowest in the lake population. Analogously, the third and fourth data columns report the number and proportion of the total tri-allelic loner SNPs (N = 368) actually being tri-allelic within each population (in parentheses the proportions are scaled as above). This latter diversity index is again lowest in the lake population.

**Population**	**Number of loner SNPs polymorphic in focal population**	**Proportion of loner SNPs polymorphic in focal population**	**Number of tri-allelic loner SNPs polymorphic in focal population**	**Proportion of tri-allelic loner SNPs polymorphic in focal population**
Lake	44,070	0.707 (100.0%)	103	0.280 (100.0%)
BOH	45,838	0.735 (104.0%)	97	0.264 (94.2%)
NID	46,632	0.748 (105.8%)	126	0.342 (122.3%)
GRA	56,280	0.903 (127.7%)	188	0.511 (182.5%)

Supplementary Table 2. Descriptive statistics for the study individuals.

Illumina library	Barcode	Specimen ID	Population	Sex	Lateral plate phenotype (l = low; p = partial; f = full)	Number of raw Illumina reads	Alignment success	Number of RAD loci yielding a consensus genotype	Mean sequence coverage across the RAD loci yielding a consensus genotype
Ga_lib_21	CGATA	Ga1085	GRA	f	p	16'123'901	0.83	287'145	43.1
Ga_lib_21	CGGCG	Ga1170	BOH	f	p	7'024'520	0.81	276'801	18.0
Ga_lib_21	CTAGG	Ga1172	BOH	f	N.A.	9'782'486	0.82	283'283	25.4
Ga_lib_21	CTGAA	Ga1426	GRA	m	p	9'224'793	0.82	284'721	24.1
Ga_lib_21	GAAGC	Ga1082	GRA	m	l	14'974'371	0.82	287'020	39.2
Ga_lib_21	GAGAT	Ga1152	BOH	f	f	4'456'312	0.82	259'253	12.1
Ga_lib_21	GCATT	Ga1173	BOH	f	N.A.	20'910'550	0.81	285'786	53.5
Ga_lib_21	GCGCC	Ga1119	UNT	m	f	27'416'119	0.78	283'349	67.5
Ga_lib_22	GGAAG	Ga1156	BOH	m	f	16'926'683	0.70	286'303	36.6
Ga_lib_22	GTACA	Ga1081	GRA	f	l	27'433'412	0.62	287'802	53.3
Ga_lib_22	TAATG	Ga1078	GRA	m	f	5'127'972	0.74	257'782	12.3
Ga_lib_22	TAGCA	Ga1110	UNT	f	p	5'164'073	0.70	266'488	12.1
Ga_lib_22	TCAGA	Ga1103	UNT	m	f	39'406'778	0.61	285'140	74.1
Ga_lib_22	TCGAG	Ga1106	UNT	f	f	2'848'858	0.71	221'902	7.6
Ga_lib_22	TGACC	Ga0312	NID	f	p	23'161'376	0.58	288'084	42.3
Ga_lib_22	TGGTT	Ga1120	UNT	m	f	20'280'307	0.54	279'849	35.0
Ga_lib_23	CGCGC	Ga0070	NID	f	l	36'334'680	0.63	274'580	73.6
Ga_lib_23	CGTAT	Ga0314	NID	m	l	23'496'158	0.64	288'445	47.9
Ga_lib_23	CTCTT	Ga1168	BOH	m	f	26'380'282	0.63	290'415	51.8
Ga_lib_23	CTTCC	Ga0087	ROM	f	f	38'114'299	0.61	276'609	73.8
Ga_lib_23	GACTA	Ga0337	NID	m	p	27'011'624	0.65	274'782	56.3
Ga_lib_23	GATCG	Ga1154	BOH	m	f	11'986'012	0.66	282'398	25.3
Ga_lib_23	GCCGG	Ga1114	UNT	m	p	11'363'641	0.67	281'197	24.2
Ga_lib_23	GCTAA	Ga0090	ROM	m	f	24'690'056	0.65	274'506	51.7
Ga_lib_24	GGCCT	Ga1155	BOH	m	l	6'711'057	0.73	252'208	16.4
Ga_lib_24	GGTTC	Ga0144	NID	f	p	30'968'137	0.67	278'899	66.1
Ga_lib_24	GTCAC	Ga1100	UNT	f	f	19'294'137	0.72	281'433	44.5
Ga_lib_24	GTTGT	Ga1427	GRA	m	l	6'193'909	0.71	262'212	14.0
Ga_lib_24	TATAC	Ga1160	BOH	m	f	25'348'324	0.70	286'704	54.5

Ga_lib_24	TCCTC	Ga0159	NID	f	f	35'699'372	0.69	279'026	79.2
Ga_lib_24	TGCAA	Ga1166	BOH	m	p	18'887'595	0.71	284'320	42.4
Ga_lib_24	TGTGG	Ga1076	GRA	m	p	6'508'538	0.69	260'893	14.7
Ga_lib_25	CGATA	Ga1104	UNT	f	f	19'778'122	0.68	284'455	42.4
Ga_lib_25	CGGCG	Ga0094	ROM	m	f	23'777'863	0.63	276'723	48.3
Ga_lib_25	CTAGG	Ga0074	NID	m	l	36'507'287	0.64	280'769	75.7
Ga_lib_25	CTGAA	Ga0228	NID	m	p	39'198'188	0.63	279'035	80.2
Ga_lib_25	GAAGC	Ga1074	GRA	m	p	14'214'345	0.68	283'572	30.2
Ga_lib_25	GAGAT	Ga1158	BOH	m	l	10'332'558	0.68	279'500	22.1
Ga_lib_25	GCATT	Ga1098	UNT	m	f	20'686'598	0.67	286'410	43.8
Ga_lib_25	GCGCC	Ga0075	NID	m	f	33'589'851	0.62	275'988	68.4
Ga_lib_26	GTACA	Ga1080	GRA	f	l	14'841'946	0.66	282'456	31.1
Ga_lib_26	CGATA	Ga0120	NID	m	p	49'283'123	0.64	277'554	102.7
Ga_lib_26	GAGAT	Ga1167	BOH	m	f	20'724'513	0.66	286'797	42.1
Ga_lib_26	TCGAG	Ga0078	NID	f	f	53'009	0.54	1'097	2.1
Ga_lib_26	ACACG	Ga0146	ROM	f	f	29'091'722	0.64	276'402	60.6
Ga_lib_26	AGAGT	Ga1107	UNT	f	f	32'492'310	0.66	285'290	67.5
Ga_lib_26	CATGA	Ga1083	GRA	f	p	8'164'759	0.72	278'025	18.4
Ga_lib_26	ATGCT	Ga0201	ROM	f	f	37'919'120	0.63	279'504	76.2
Ga_lib_27	TAATG	Ga0114	ROM	m	p	24'970'277	0.60	289'170	45.8
Ga_lib_27	CGATA	Ga1084	GRA	m	l	13'537'909	0.63	283'001	26.7
Ga_lib_27	CATGA	Ga0122	NID	f	f	18'837'281	0.64	273'140	39.7
Ga_lib_27	GAGAT	Ga1163	BOH	m	l	17'815'656	0.53	286'542	29.3
Ga_lib_27	GTCAC	Ga0077	NID	f	f	13'431'633	0.59	273'935	25.9
Ga_lib_27	AGAGT	Ga0336	NID	m	p	33'777'653	0.60	277'001	64.9
Ga_lib_27	ATGCT	Ga0286	ROM	m	p	35'918'356	0.57	284'520	63.7
Ga_lib_27	TCGAG	Ga1079	GRA	m	f	11'471'522	0.61	285'134	21.8
Ga_lib_28	TAGCA	Ga1077	GRA	m	l	24'159'978	0.60	261'321	46.1
Ga_lib_28	GGAAG	Ga1165	BOH	m	p	11'467'443	0.65	198'943	29.9
Ga_lib_28	TCGAG	Ga0082	NID	f	l	28'028'659	0.63	261'961	57.9
Ga_lib_28	GTCAC	Ga0099	ROM	m	f	20'048'656	0.66	245'333	45.6
Ga_lib_28	ACACG	Ga1431	GRA	f	l	7'167'002	0.65	151'191	24.0
Ga_lib_28	AGTCA	Ga0121	NID	m	l	24'008'515	0.60	260'951	46.2
Ga_lib_28	CATGA	Ga1118	UNT	f	f	16'869'124	0.55	256'994	29.2
Ga_lib_28	CGATA	Ga0293	ROM	f	f	36'304'322	0.59	267'543	68.2
Ga_lib_29	TCAGA	Ga1429	GRA	f	f	38'662	0.45	427	2.1

Ga_lib_29	TTCCG	Ga0157	ROM	m	f	30'258'306	0.49	285'983	45.3
Ga_lib_29	AGTCA	Ga1164	BOH	m	p	41'196'483	0.60	288'512	77.4
Ga_lib_29	ATGCT	Ga0160	NID	f	l	35'216'672	0.58	287'895	62.5
Ga_lib_29	CGATA	Ga0073	NID	f	p	34'578'247	0.57	286'229	62.1
Ga_lib_29	GAGAT	Ga1424	GRA	f	l	5'868'636	0.58	264'874	10.8
Ga_lib_29	GTCAC	Ga1420	GRA	f	l	6'131'229	0.55	264'470	10.6
Ga_lib_29	TGTGG	Ga1171	BOH	f	f	1'515'400	0.56	155'540	4.0
Ga_lib_30	TCGAG	Ga1125	UNT	m	f	61'344	0.70	6'371	2.5
Ga_lib_30	TTCCG	Ga1430	GRA	f	f	19'369'795	0.74	289'519	43.5
Ga_lib_30	ACACG	Ga1161	BOH	m	f	24'148'648	0.74	285'231	57.0
Ga_lib_30	AGTCA	Ga0313	NID	m	f	52'994'326	0.70	281'502	118.5
Ga_lib_30	GTCAC	Ga1425	GRA	f	l	28'300'487	0.71	283'839	63.3
Ga_lib_30	ATATC	Ga0233	NID	m	p	23'387'686	0.74	288'891	54.3
Ga_lib_30	CGATA	Ga1157	BOH	f	f	28'724'046	0.74	286'499	64.9
Ga_lib_31	TGACC	Ga1075	GRA	m	l	104'698	0.29	762	2.1
Ga_lib_31	TCGAG	Ga0081	NID	f	p	24'323'290	0.47	240'663	37.6
Ga_lib_31	ACTGC	Ga1162	BOH	m	f	4'933'837	0.56	226'296	9.3
Ga_lib_31	AGTCA	Ga0234	NID	m	l	22'305'914	0.54	252'085	40.0
Ga_lib_31	CATGA	Ga1418	GRA	f	l	4'870'606	0.58	229'118	9.4
Ga_lib_31	GAGAT	Ga0145	ROM	f	l	16'475'686	0.48	239'448	26.5
Ga_lib_31	GTCAC	Ga1099	UNT	m	f	14'728'702	0.56	257'067	26.8
Ga_lib_31	ATATC	Ga1169	BOH	m	f	3'332'506	0.59	206'238	6.9
Ga_lib_32	TGGTT	Ga1159	BOH	m	f	53'619	0.32	357	2.1
Ga_lib_32	TATAC	Ga1422	GRA	f	l	33'707'043	0.58	275'175	64.6
Ga_lib_32	CGATA	Ga0232	NID	m	p	23'694'642	0.46	273'891	35.6
Ga_lib_32	GAGAT	Ga1153	BOH	m	l	25'959'379	0.62	287'120	50.4
Ga_lib_32	GTCAC	Ga0084	NID	f	f	17'661'738	0.61	270'380	35.1
Ga_lib_32	ACTGC	Ga1073	GRA	m	p	7'825'103	0.64	273'226	15.7
Ga_lib_32	AGTCA	Ga1436	ROM	f	p	27'512'614	0.49	271'700	44.3
Ga_lib_32	CATGA	Ga1421	GRA	m	f	7'438'624	0.58	270'517	13.7

SUPPLEMENTARY REFERENCES

1. Moser, D., Roesti, M. & Berner, D. Repeated lake-stream divergence in stickleback life history within a Central European lake basin. *PLoS One* **7**, e50620 (2012)
2. Jones, F. C. et al. The genomic basis of adaptive evolution in threespine sticklebacks. *Nature* **484**, 55-61 (2012).
